# Quantitative Genome-Wide Genetic Interaction Screens Reveal Global Epistatic Relationships of Protein Complexes in *Escherichia coli*

**DOI:** 10.1371/journal.pgen.1004120

**Published:** 2014-02-20

**Authors:** Mohan Babu, Roland Arnold, Cedoljub Bundalovic-Torma, Alla Gagarinova, Keith S. Wong, Ashwani Kumar, Geordie Stewart, Bahram Samanfar, Hiroyuki Aoki, Omar Wagih, James Vlasblom, Sadhna Phanse, Krunal Lad, Angela Yeou Hsiung Yu, Christopher Graham, Ke Jin, Eric Brown, Ashkan Golshani, Philip Kim, Gabriel Moreno-Hagelsieb, Jack Greenblatt, Walid A. Houry, John Parkinson, Andrew Emili

**Affiliations:** 1 Banting and Best Department of Medical Research, Donnelly Centre, University of Toronto, Toronto, Ontario, Canada; 2 Department of Biochemistry, Research and Innovation Centre, University of Regina, Regina, Saskatchewan, Canada; 3 Hospital for Sick Children, Toronto, Ontario, Canada; 4 Department of Biochemistry, University of Toronto, Toronto, Ontario, Canada; 5 Department of Molecular Genetics, University of Toronto, Toronto, Ontario, Canada; 6 Department of Biochemistry and Biomedical Sciences, McMaster University, Hamilton, Ontario, Canada; 7 Department of Biology and Ottawa Institute of Systems Biology, Carleton University, Ottawa, Ontario, Canada; 8 Department of Biology, Wilfrid Laurier University, Waterloo, Ontario, Canada; University of Massachusetts, United States of America

## Abstract

Large-scale proteomic analyses in *Escherichia coli* have documented the composition and physical relationships of multiprotein complexes, but not their functional organization into biological pathways and processes. Conversely, genetic interaction (GI) screens can provide insights into the biological role(s) of individual gene and higher order associations. Combining the information from both approaches should elucidate how complexes and pathways intersect functionally at a systems level. However, such integrative analysis has been hindered due to the lack of relevant GI data. Here we present a systematic, unbiased, and quantitative synthetic genetic array screen in *E. coli* describing the genetic dependencies and functional cross-talk among over 600,000 digenic mutant combinations. Combining this epistasis information with putative functional modules derived from previous proteomic data and genomic context-based methods revealed unexpected associations, including new components required for the biogenesis of iron-sulphur and ribosome integrity, and the interplay between molecular chaperones and proteases. We find that functionally-linked genes co-conserved among γ-proteobacteria are far more likely to have correlated GI profiles than genes with divergent patterns of evolution. Overall, examining bacterial GIs in the context of protein complexes provides avenues for a deeper mechanistic understanding of core microbial systems.

## Author Summary

Genome-wide genetic interaction (GI) screens have been performed in yeast, but no analogous large-scale studies have yet been reported for bacteria. Here, we have used *E. coli* synthetic genetic array (eSGA) technology developed by our group to quantitatively map GIs to reveal epistatic dependencies and functional cross-talk among ∼600,000 digenic mutant combinations. By combining this epistasis information with functional modules derived by our group's earlier efforts from proteomic and genomic context (GC)-based methods, we identify several unexpected pathway-level dependencies, functional links between protein complexes, and biological roles of uncharacterized bacterial gene products. As part of the study, two of our pathway predictions from GI screens were validated experimentally, where we confirmed the role of these new components in iron-sulphur biogenesis and ribosome integrity. We also extrapolated the epistatic connectivity diagram of *E. coli* to 233 distantly related γ-proteobacterial species lacking GI information, and identified co-conserved genes and functional modules important for bacterial pathogenesis. Overall, this study describes the first genome-scale map of GIs in gram-negative bacterium, and through integrative analysis with previously derived protein-protein and GC-based interaction networks presents a number of novel insights into the architecture of bacterial pathways that could not have been discerned through either network alone.

## Introduction

A key feature of the molecular organization of microbes is the tendency of functionally-linked proteins to associate as components of macromolecular complexes, operons, or other biological groupings. As a consequence, the gene products present in a bacterial cell are organized into functional modules, which in turn mediate the major cellular pathways and processes that support bacterial cell growth, proliferation, and adaptation [1]–[3]. Identifying the pairwise functional relationships between genes can reveal these modules, and elucidate the molecular systems that underlie the functional organization of a microbial cell. While chromosomal associations informative about gene functional relationships can be inferred computationally using genomic context (GC)-based methods [4], [5], knowledge of the composition and connectivity of multiprotein complexes and their organization into pathways requires experimentation, and such information remains incomplete even in one of the most tractable and well-studied, prokaryotic model-organisms, *Escherichia coli* [1], [6].

Physical interactions can be mapped with high-confidence based on the affinity-purification of chromosomally-tagged proteins in combination with mass spectrometry (APMS), which aims to isolate and identify endogenous protein complexes. Analogous to the tandem affinity purification (i.e., TAP tag) method developed for yeast [7]–[9], we developed an efficient sequential peptide affinity purification procedure for *E. coli* [2], [10] and used it to decipher the global physical organization of a bacterial cell [2], [10]–[12]. Our protein-protein interaction (PPI) map allows for the prediction of protein functions for previously uncharacterized components of soluble macromolecular complexes that co-purify with functionally annotated subunits, via ‘guilt-by-association’ [2], [10]. We further integrated our proteomic data with comparative genomic inferences to define a more comprehensive network of functional interactions covering most of *E. coli*'s cytosolic proteome [2], [3]. Nevertheless, these maps do not fully capture the global systems organization of complexes within biological pathways or processes.

To this end, we and others have developed high-throughput genetic screening methods to systematically map epistasis relationships (i.e., genetic interactions, abbreviated as GIs hereafter) between bacterial gene pairs [13]–[16]. Biochemical pathways and networks are often robust [17], such that most bacterial genes produce no discernible phenotype when singly deleted or mutated [18]. Indeed, only ∼300 of *E. coli's* 4,145 protein-coding genes are essential under standard laboratory conditions [19]. However, examining the fitness of double mutants can reveal functional dependencies. Hence, our quantitative *E. coli* synthetic genetic array (eSGA) technology, which simplifies the systematic generation and phenotypic scoring of large numbers of double mutants created by mating collections of engineered *E. coli* strains en masse [13], [16], can reveal the functional relationships of previously uncharacterized gene products [1], [6]. For example, loss of two non-essential genes, which functionally compensate or buffer each other, may show an aggravating (synthetic sick or lethal, or SSL) GI if the combination of mutations critically impairs a process essential for cell growth or viability. Conversely, ‘alleviating’ (i.e., buffering or suppression) GIs can occur between two genes encoding subunits of the same protein complex, where inactivation of either one alone annihilates complex activity, such that loss of the second component confers no additional defect. Indeed, the global patterns of aggravating and alleviating interactions measured by large-scale GI screens have been used to decipher the functional organization of biological pathways and protein complexes in yeast [20]–[23].

Here, to study the global organization of the *E. coli* interactome, we employ our eSGA approach in an unbiased manner by performing 163 functionally diverse query genes. The resulting filtered GI network was then combined with existing PPI data and GC-derived interactions to reveal pathway-level crosstalk between disparate protein complexes, and specific biological roles of uncharacterized bacterial gene products.

## Results

### Target gene selection for an unbiased GI survey

**Figure 1:**
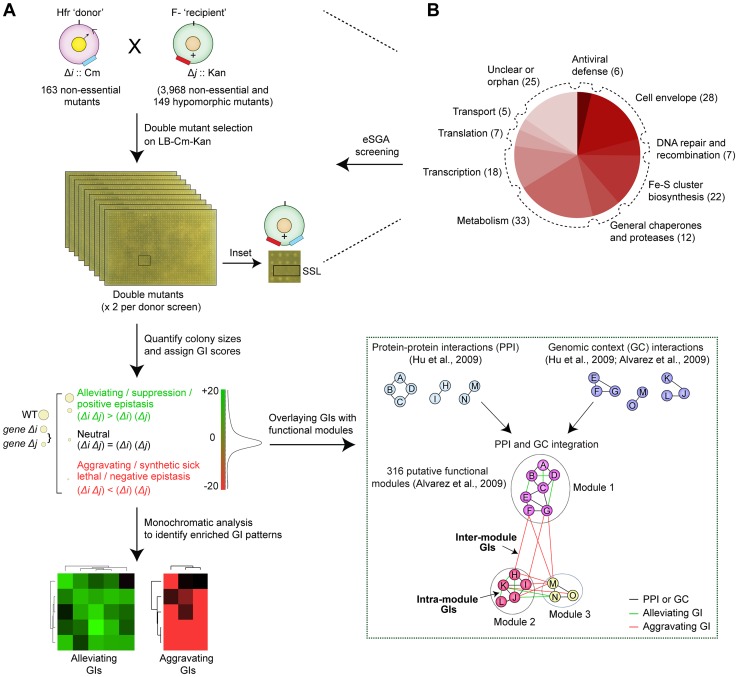
Target selection and eSGA screen pipeline. (A) Schematic showing conjugation-based double mutant construction, colony imaging, and fitness scoring [13], [16]. The GIs were subjected to monochromatic analysis [45] to identify functionally related gene groups with similar GI patterns and overlaid with putative functional modules defined from PPI and GC-based networks [2], [3]. (B) Bioprocess annotations and numbers (parenthesis) of functionally divergent query genes subjected to genome-wide eSGA screens.

Since fully comprehensive screens are not yet practicable, we selected a diverse, minimally-redundant set of broadly representative ‘query’ genes for our screens (see Protocol S1). After generating selectable mutants in a hyper-recombinant Hfr-Cavalli (Hfr C) ‘donor’ strain background marked with a chloramphenicol-resistance cassette (Cm^R^), the corresponding deletion alleles were transferred by conjugation into a near genome-wide mutant collection of F- ‘recipient’ mutant strains, arrayed in duplicate at 384-colony density. This collection, contains 3,968 non-essential single gene deletions in which the open reading frame was replaced and marked by a kanamycin resistance (Kan^R^) cassette (i.e., the Keio collection) [19], and 149 hypomorphic mutant strains [13], [16], in which a Kan^R^ marker was integrated into the 3′-UTR to alter transcript abundance or stability [13] (Figure 1A, Protocol S2).

**Figure 2:**
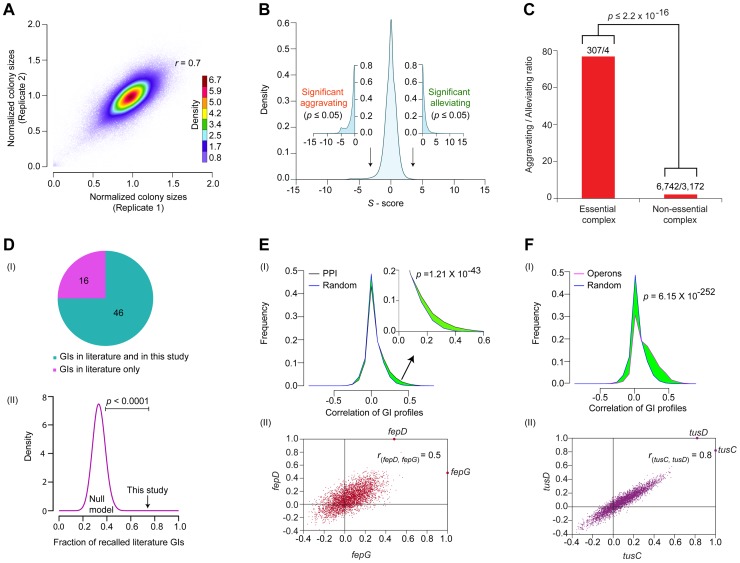
Functional properties of the global *E. coli* GI network. (A) Reproducibility of normalized colony sizes of digenic mutants measured in replicate screens. (B) Histogram of GI *S*-scores; arrows indicate cut-off scores (|*S*- score±3|; *p*-value ≤0.05 computed using Fisher's exact test) used to signify significant epistatic (aggravating or alleviating) interactions. (C) Comparison of aggravating-to-alleviating GI ratios observed among essential and non-essential complex components. Numbers represent the total aggravating over alleviating GIs in essential or non-essential complexes. (D) Overlap of GI compared to literature in terms of (I) coverage and (II) statistical significance (black arrow) versus background frequencies generated by random permutation (purple distribution represents 10,000 random null models). Distributions of GI correlation profiles (I) of genes either (E) encoding physically interacting proteins (zoom-in of right tail shown in inset) or (F) within same operon versus randomly drawn gene pairs; significance values computed using two-sample Kolmogorov-Smirnov (KS) test. (II) Representative scatter plots show correlated GI profiles of *fepD* (*y*-axis) vs. *fepG* (*x*-axis), and *tusC* (*x*-axis) vs. *tusD* (*y*-axis).

In total, a set of 163 query ‘donor’ genes with evidence of expression and whose products had high physical interaction degree were selected for screening (Protocol S1). These included 93 genes linked to core bacterial processes (Figure 1B), such as metabolism, cell envelope biogenesis, transcription, protein synthesis and chromosomal replication and repair, and 25 genes of unknown function (Table S1). Since accurate quantitation of epistasis depends on reliable estimations of mutant fitness [24], we performed two independent replicate screens such that each donor-recipient mutant gene pair was tested eight times to account for experimental variation (see Protocol S2). Following genetic transfer, the double mutants were selected on rich medium (Luria Broth) containing both marker drugs (Kan+Cm). After outgrowth for 36 hrs at 32°C, the plates were imaged digitally. Colony growth was quantified using a data processing strategy originally devised for yeast SGA analysis [24], [25], to correct for possible batch and plate position effects, and the different intrinsic growth rates of the single mutants [26]. We also eliminated from consideration pairs of closely-linked loci that potentially suffer from reduced recombination efficiency due to linkage suppression [24], [25]. Overall replicate screen reproducibility was high (*r* = 0.7; Figure 2A), similar to that reported for other high-quality GI studies [16], [24], [27].

### Generating a genome-wide network of high-confidence GIs

We used a multiplicative model to calculate epistasis (*S*) scores [21], [22], [28], determining both the strength and confidence of putative GIs based on differences between the observed growth of the digenic mutants and the expected growth rates. The null hypothesis assumes independent fitness defects for non-interacting gene pairs - that is, if two alleles are functionally unrelated (i.e., independent), their joint fitness defects should combine in a multiplicative (i.e., non-synergistic) manner, as was done previously for yeast [25], [29]. Conversely, *S*-scores deviating significantly from expectation represent candidates for functional associations (i.e., genes working together in a pathway to perform a specialized cellular activity) [29].

The *S*-scores calculated for ∼600,000 digenic mutant combinations tested showed a normal distribution centered on zero (i.e., neutral) (Figure 2B), consistent with the expectation that GIs are relatively rare, with the fitness of most double mutants (i.e., functionally unrelated) typically equal to the product of individual single mutant growth defects [1], [30]. To rigorously define GIs, as with our previous studies [13], [16], we applied stringent statistical thresholds corresponding to two standard deviations (|Z-score|≥2; *P*≤0.05) of the score distribution to define significant outliers (Protocol S2). After filtering, the network encompassed GI with *S*-scores of −3 or lower (25,239 in total) that indicate aggravating (i.e., SSL) relationships, and GIs with *S*-scores of +3 or higher (17,466) representing alleviating relationships (Figure 2B, Table S2), which occasionally (but rarely) reflect suppression of an impaired growth phenotype conferred by a single allele.

Like other biological networks [24], [31], the filtered GI network had a modular connectivity structure (average clustering coefficient = 0.23, Figure S1A), wherein the majority of the genes have few GIs compared to a small number (n = 25) of highly connected (edge ≥640) ‘hubs’ (Figure S1B). As was reported for yeast [27], [32], [33], essential *E. coli* genes tend to be more highly connected in the network compared to non-essential genes, both in terms of GI degree (Figure S1C, Protocol S3) and overall network betweenness (i.e., a graph centrality measure reflecting the proportion of shortest paths between pairs of nodes that go through a particular gene) (Figure S1D, Protocol S3). Essential subunits of annotated protein complexes are also significantly enriched (*p* = 2.2×10^−16^) in aggravating interactions with each other, compared to pairs of components within non-essential complexes (Figure 2C, Protocol S3), suggesting that as in yeast [34], essential bacterial protein complexes occupy a central position within the *E. coli* GI network, just as they do in the *E. coli* PPI network [10].

### External benchmarking

Comparison of the filtered GI network against a reference set of manually curated GIs extracted from the literature showed high (∼75%) agreement, which is significant (*p*-value ≤10^−4^) by random sampling null models (Figure 2D, Table S3, Protocol S3). For instance, our network captured the synthetic lethality reported between mutants of the chaperones, *cbpA* and *dnaJ* [35], and between the exonucleases *recB* and *recJ*, and *recB* and components of the RecFOR DNA repair complex, which jointly function in RecA-mediated recombination [36].

As the number of interactions in the literature curated reference set was quite limited, we examined if the interacting gene pairs were enriched for functional relatedness using a battery of different metrics (see Protocol S4). For example, inspection of the GI network revealed a slight but significant (*p* = 1.2×10^−43^) tendency for *E. coli* genes encoding subunits of the same protein complexes to display correlated patterns of GIs as compared to randomly selected protein pairs (Figure 2E). Likewise, the components of the membrane-associated ferric enterobactin permease complex, FepD and FepG [37], [38], showed highly correlated (*r_fepD,fepG_* = 0.5; Figure 2E) GI patterns, consistent with their co-operative role in transporting iron-bound siderophores into the cytoplasm [39].

Indeed, by every other measure examined, including functional associations predicted by GC methods (*p* = 2.2×10^−118^) [2], mRNA co-expression (*p* = 3.3×10^−93^) [40], and phenomic (i.e., chemical genetic, *p* = 4.8×10^−14^) profiles [41]; we found that pairs of genes showing similar connectivity patterns in the GI network tended to be more highly correlated (i.e., as measured by Pearson Correlation Co-efficient (PCC) scores) (Figure S2A–C, Protocol S4). Similarly, genes present within the same operon in *E. coli* [42] had significantly (*p* = 6.1×10^−252^) more positively correlated genetic profiles than random pairs of genes (Figure 2F), and this correlation was likely not due to polarity effects as the last and the first genes within each operon were, on average, just as likely to be positively correlated as the first and the middle genes (Figure S2D); intuitively, however the last gene cannot possibly underlie the GI phenotypes for every operon (Protocol S5). An illustrative example is the highly similar (*r_tusC, tusD_* = 0.8) GI patterns of the two gene products, *tusCD*, encoded by the sulfur mediator operon, *tusBCDE* (Figure 2F), consistent with their joint role in coordinating sulfur transfer [43]. Taken together, the benchmarking underscored the reliability and coverage of our screen data, indicating that the filtered GI network is informative about biological relationships at the level of individual gene pairs, multiprotein complexes, and pathways.

### Probing functional neighborhoods in GI networks by monochromaticity

**Figure 3:**
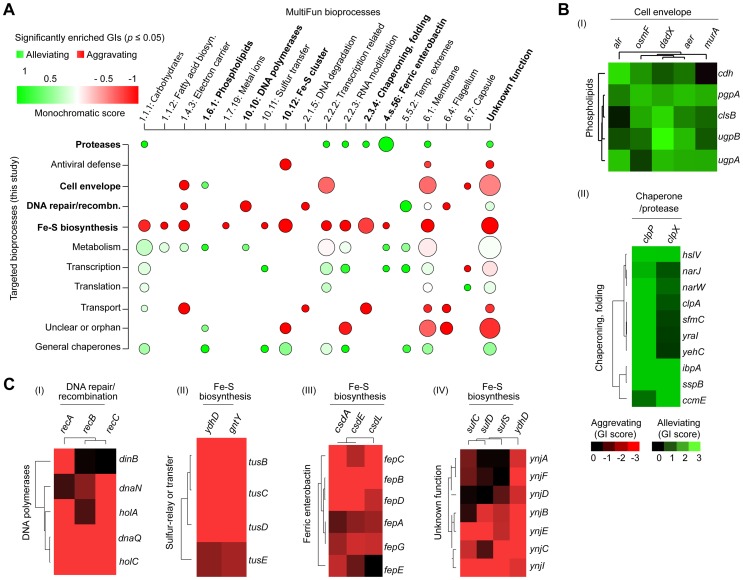
Monochromaticity of GIs among bacterial bioprocesses. (A) Heatmap displaying the distribution of significantly enriched (*p*-value ≤0.05) aggravating or alleviating GIs between functional categories. Node size represents the number of enriched GIs per process, while the color indicates the monochromaticity type: red for aggravating (monochromatic score of −1) and green for alleviating (monochromatic score of +1). Only representative MultiFun processes (x-axis) are shown. Highlighted (bold) crosstalk processes are shown as separate sub-networks in panels B and C. Heatmaps showing overlapping patterns of alleviating (B) or aggravating (C) GIs for representative genes within particular categories after hierarchical clustering.

To identify broader functional groupings (i.e., modules or interconnected gene sets), we sorted the genes according to their biological process annotations, and examined the extent to which their corresponding high-confidence GI (|*S*-score≥3|; *P*≤0.05) tended towards alleviating or aggravating GI (Figure 3A), using a “monochromatic” score that has been previously used to unveil the modularity of yeast GI networks [44], [45]. While discrete clusters were clearly identified (Figure 3B and 3C) from the GI spanning the constituent genes within bioprocesses with high alleviating or aggravating monochromatic scores, several of these bioprocesses displayed extensive inter-connectivity, suggestive of biological cross-talk (Table S4, Protocol S6). For example, alleviating interactions bridge the cell envelope machinery (e.g., *alr*, *dadX*, *aer*) to phospholipid biosynthesis (*clsB*, *pgpA*, *ugpA*, *ugpB*, *cdh*) (Figure 3B), consistent with their close coupling during membrane formation and integrity [16], [46].

Conversely, other process combinations were preferentially enriched for aggravating relationships (Figure 3C). For example, strong SSL associations were observed between the homologous recombination machinery (*recABC*) and DNA polymerases [polIII (*dnaNQ*, *holAC*); polIV (*dinB*)], whose coordination is critical for genomic integrity [47]. Likewise, sulfur-relay systems [*yccK* (*tusE*), *yheLMN* complex (*tusBCD*)], which channel sulfur from various trafficking pathways to 2-thiouridine [43], showed aggravating interactions with downstream iron-sulfur (Fe-S) cluster scaffold assembly factors (e.g., *ydhD*, *gntY*) (Figure 3C). Similarly, the ferric (Fe^3+^) enterobactin transporter system (e.g., *fepBCDG* complex, *fepA*, *fepE*) showed strong SSL links with the CSD (cysteine sulfinate desulfinase) sulfur transfer apparatus (e.g., *csdAEL*) (Figure 3C), implying overlap in iron homeostasis.

### Functional insights revealed by unexpected epistatic pathway relationships

Since the global patterns of GI measured by eSGA reflect biological relationships, we examined our GI network specifically to delineate novel functional roles for bacterial genes of unclear biological significance. Clustering the GIs resulting from the monochromatic analysis (Protocol S6) implicated orphan genes lacking annotations to specific pathways. For instance, seven unannotated genes (*ynjABCDEFI*) were grouped together with particular components (e.g., *sufCDS*, *ydhD*) of the “Suf” Fe-S cluster assembly machinery (Figure 3C), consistent with a recent report that YnjE is a sulfur transferase required for molybdopterin biosynthesis [48].

**Figure 4:**
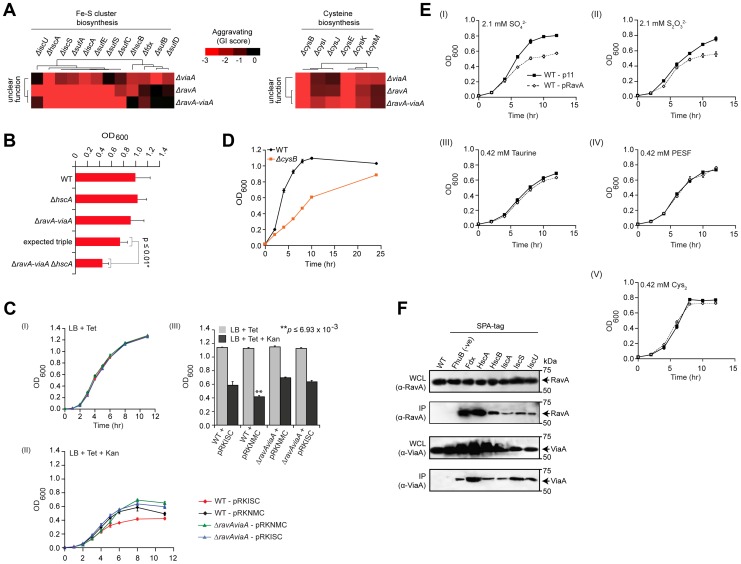
RavA and ViaA linked to Fe-S assembly. (A) Sub-network of GIs of two unannotated genes with Fe-S cluster assembly and cysteine biosynthesis components. (B) Differential growth of select single, double and triple mutants in rich medium (LB) at 32°C over 24 h; expected fitness derived using multiplicative model, *p*-value calculated using Student's *t*-test. (C) Impact of ectopic over-expression of Isc Fe-S cluster assembly proteins (pRKISC expression plasmid vs. pRKNMC control empty vector) on growth of *ravA-viaA* double mutants vs. wild-type (WT) *E. coli* before (I) and after (II) oxidative stress (sub-lethal concentrations of kanamycin, Kan); OD_600_ readings at 11-hr time point (III) highlight differential responses. Tetracycline (Tet) included in media for plasmid maintenance. Asterisks represent significant (*p*≤0.01; Student's *t*-test) difference between WT+ pRKISC vs. WT+ pRKNMC. (D) Slow growth of *cysB* deletion mutants on liquid LB medium at 32°C. Each data point shows the mean ± SD (error bars) of three independent biological measurements. (E) Growth inhibition profiles of ectopic over-expression of *ravA* (pRavA) vs. WT (p11) on W-salt medium supplemented with sub-lethal concentration of inorganic (I and II) and organic (III–V) sources of sulphur. (F) Co-immunoprecipitation analysis of endogenous RavA (top) and ViaA (bottom). Immunoblots show chromosomally tagged Isc assembly proteins, expressed at native levels, in input whole cell lysate (WCL) and anti-FLAG immunoprecipitates (IP) as indicated. Untagged parental strain and an irrelevant bait protein (ATP-dependent iron hydroxamate transporter, FhuB), served as negative controls. Molecular masses (kDa) of marker proteins by SDS-PAGE are indicated.

Another illustrative example is a modular sub-network consisting of RavA (Regulatory ATPase variant A), a AAA+ ATPase of the MoxR protein family whose physiological function is uncertain, and its binding partner, ViaA (von Willebrand factor A domain interacting AAA+ ATPase) [49], which also exhibited strong aggravating connections with the Fe-S cluster assembly apparatus (Figure 4A). Consistent with predicted epistasis, *ravA viaA Fe-S* triple deletion strains showed virtually identical GIs (i.e., SSL) as *ravA Fe-S* or *viaA Fe-S* double mutants (Figure 4A), which were confirmed independently by liquid culture growth assays (Figure 4B, Protocol S7; representative *ravA viaA hscA* triple mutant shown).

To further examine the link with Fe-S assembly, we exploited the observations that, at sub-lethal dosages, bactericidal drugs such as aminoglycosides (e.g., streptomycin, gentamycin) cause cell death via mechanisms that are dependent on Fe-S clusters [50]–[53], and that the uptake of aminoglycosides are directly influenced by the Isc pathway of Fe-S cluster biogenesis [54]. As a result, strains deficient in Fe-S assembly show decreased drug sensitivity [52], [54]. We therefore tested the influence of *ravA* and *viaA* on Fe-S biogenesis in strains over-expressing the *isc* assembly machinery (*iscRSUA*-*hscBA*-*fdx-iscX*) on a multicopy plasmid (pRKISC) [55] upon challenge with the aminoglycoside, kanamycin. Notably, the presence of kanamycin impaired wild-type, but not *ravA viaA* double mutants (Figure 4C, Protocol S8).

Consistent with this, *ravA* and *viaA* also showed GIs with cofactors required for Fe-S cluster formation, including genes involved in the biosynthesis of L-cysteine (e.g., the serine acetyltransferase complex, *cysEK*; hemoprotein subunit of sulfite reductase, *cysIJ*) from which precursor sulfur is extracted (Figure 4A). The fact that cysteine biosynthetic genes become essential despite the presence of rich media suggests a defect in cysteine transport in the *cysB* mutant strain (Figure 4D, Protocol S7). Thus, defects in the de novo biosynthesis of cysteine, coupled with impaired import, likely decrease the pool of cysteine available for Fe-S biogenesis and related sulfur transfer reactions by this pathway, which is mirrored as an aggravating phenotype. Since the uptake and assimilation of inorganic sulfurs by cysteine biosynthesis genes in bacteria requires the CysAUWP ABC transporter complex [56]–[58], while organic sulfurs are imported by other ABC transporters [59], we challenged strains over-expressing *ravA* with inorganic (e.g., SO_4_^2−^ and S_2_O_3_^2−^) and organic [taurine, 2-(4-pyridyl)-ethanesulfonate (PESF), and cysteine (i.e., Cys-S-S-Cys)] sulfur compounds (Figure 4E, Protocol S9). Unlike wild-type *E. coli*, *ravA* over-expressing strains showed increased sensitivity to inorganic, but not organic sulfurs (Figure 4E, Protocol S9), seemingly due to perturbation of the normal RavA-ViaA stoichiometry necessary for normal cell function. Taken together, a direct or indirect impact of RavA/ViaA on bacterial sulfur transport is consistent with our GI data, reflecting the tight integration of these systems.

Since the growth assays confirmed participation of *ravA* and *viaA* in Fe-S assembly (Figure 4B and 4C), we performed co-immunoprecipitation (co-IP) experiments to determine whether these two proteins interact physically with the Fe-S cluster (Isc) assembly proteins, with which they showed strong aggravating interactions (Figure 4A). Indeed, endogenous affinity-tagged Isc proteins specifically and efficiently co-precipitated native RavA and ViaA (Figure 4F, Protocol S10), implying joint participation in cellular iron homeostasis through physical associations. Most notably, the fact that *ravA*-*viaA* mutants displayed a strong aggravating phenotype between the subunits of Isc complex supports the idea that these two overlooked processes function redundantly to tightly regulate cellular iron levels required for the maintenance of cell viability. That is while deletion of subunits of either protein complex shows a similar effect as loss of the entire complex, mutations in both complexes (i.e., RavA-ViaA and Isc simultaneously perturbed) result in SSL phenotypes due to system failure.

**Figure 5:**
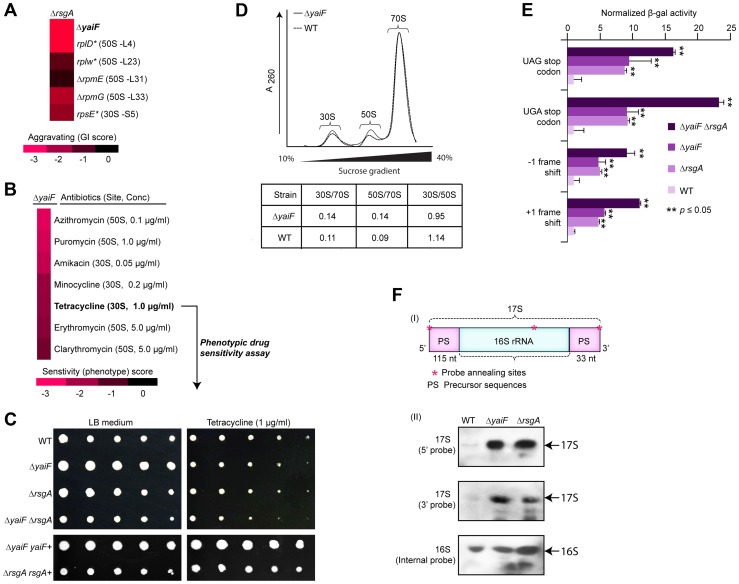
YaiF linked to ribosome biogenesis. (A) Aggravating GIs between *yaiF* and 30S subunit biogenesis factor, *rsgA*, and components of the 30S (*rpsE*) and 50S (*rplD*, *rplW*, *rpmE*, *rpmG*) ribosomes. (B) Drug hypersensitivity of a *yaiF* deletion strain to antibiotics targeting the ribosome/translational reported in a recent chemical-genetic screen [41]. Drug concentration producing a significant phenotype is indicated in parentheses. (C) Sensitivity of *yaiF* and *rsgA* single and double mutants versus wild-type cells (WT) to tetracycline (1.0 µg/ml). Panel below shows phenotypic complementation by over-expression *in trans*. (D) Different ribosome profiles in *yaiF* deletion mutant vs. WT strains. Quantification of ribosome subunit peak ratios is provided. (E) Increased translational errors, based on read-through of a β-galactosidase reporter (normalized to a control vector), in *yaiF* and *rsgA* single and double mutants relative to WT cells. Asterisks indicate significant (Student's *t*-test) difference between single or double mutant vs. WT strains. (F) Schematic showing the precursor sequences (PS) of the 17S rRNA (I) with oligonucleotide probe annealing (shown as asterisks) sites. The 115 and 33 nucleotides shown in the 5′ and 3′ ends of the 17s rRNA is the precursor rRNA for 30S ribosomal subunit [107]. Northern hybridization shows the accumulation of 17S rRNA species in mutants and WT strains (II) using the indicated biotinylated oligonucleotide probes. The 16S rRNA probe was used as an internal control.

Another example of functional insights resulting from this GI analysis involves a sub-network (Figure 5A) of aggravating GIs connecting the late ribosome biogenesis factor, *rsgA*, with both the ribosome and an unannotated gene, *yaiF*, which, while not essential in *E. coli*, is predicted to belong to a protein family of acetyl-transferases that are widely conserved among microbes (Table S5). Although the co-IP experiments showed no physical association between YaiF and RsgA in *E. coli* solubilized cell extracts (data not shown), as with the GI dataset, analysis of previously published large-scale phenomics (i.e., chemical genetic profiling) data [41] showed that a mutant strain lacking *yaiF* is hyper-sensitive to antibiotics (macrolide, tetracycline, amino-glycoside) targeting protein synthesis (Figure 5B). Similarly, we found that the mutant strain lacking *yaiF* or *rsgA* was sensitive to tetracycline, whereas the *yaiF rsgA* double mutant exhibited increased drug sensitivity (Figure 5C, Protocol S7), suggesting participation of YaiF in translation.

To evaluate this link further, we examined ribosome profiles in *yaiF* deletion mutants. Unlike *rsgA*, the ribosome profile of *yaiF* mutant from the log-phase culture was nearly wild-type (Figure 5D, Protocol S11), consistent with the previous finding where loss of known protein synthesis gene products, including the ribosome modulation factor, *rmf* [60], resulted in near wild-type profiles. However, in contrast to wild-type cells, *yaiF* or *rsgA* mutants exhibited translational defects, including mistranslation as indicated by higher read-through of out-of-frame amber (UAG) and opal (UGA) nonsense codon alleles and miscoding of +1 and −1 frame-shift mutations in a β-galactosidase reporter [61] (Figure 5E, Protocol S11). Strikingly, these defects were exacerbated when both *yaiF* and *rsgA* were deleted (Figure 5E, Protocol S11), consistent with our genetic data.

Moreover, strains lacking *yaiF* delayed the production of mature 16S rRNA, resulting in the accumulation of late unprocessed 17S rRNA molecules (Figure 5F, Protocol S11) in a similar manner to the mutant strain lacking *rsgA* [62], [63]. This effect was specific as overexpression of *yaiF* or *rsgA in trans* fully rescued the 17S rRNA defects in the respective deletion strains (data not shown), indicating the involvement of YaiF in bacterial protein synthesis. However, further experiments are warranted to delineate how YaiF affects RNA processing and ribosome biogenesis, potentially in a pathway relating to RsgA.

### Genetic networks showcase the systems coupling supporting protein homeostasis

**Figure 6:**
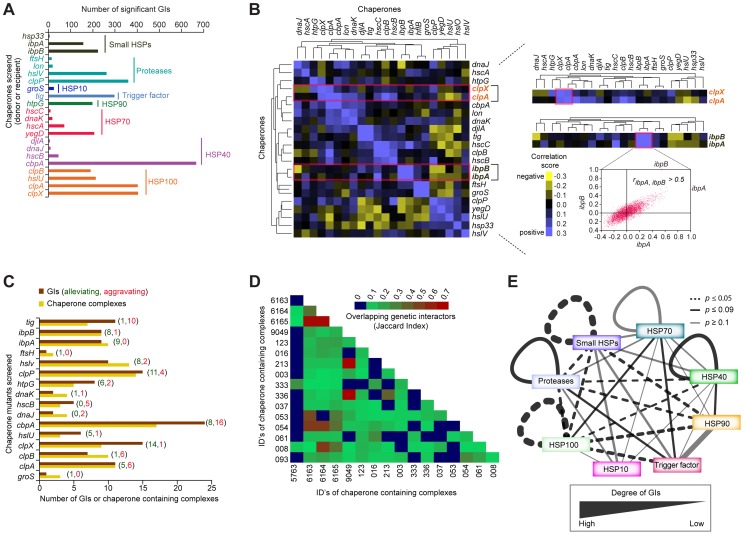
Functional crosstalk among chaperones and proteases. (A) Summary of chaperone type and GI frequency observed by eSGA. (B) Heatmap showing clusters of correlated GI profiles among select chaperones. Highlighted sub-networks show similar (correlated) GI profiles between the ATP-dependent protein unfoldases *clpX* and *clpA* (top), and the small HSPs *ibpA* and *ibpB* (bottom). Scatter-plot shows genome-wide correlation coefficient profiles of *ibpA* (*x*-axis) versus *ibpB* (*y*-axis). (C) Number of alleviating (green) or aggravating (red) GIs of each chaperone mutant (brown bar) with one or more chaperone-containing protein complexes (orange bar), compiled from Ecocyc and our own previous work [2]. (D) Shared (jaccard index) non-chaperone interactors among chaperone-containing protein complexes. (E) Crosstalk among chaperone and protease families. Edge thickness represents degree of GI connectivity within and between families; dark edges indicate statistically significance (*p*-value ≤0.09; hypergeometric test).

Molecular chaperones often have numerous binding partners, as they typically participate in the folding, assembly, transport, and stability of multiple client proteins involved in distinct processes [64], [65]. Previous systems-wide analyses of physical and genetic interactions involving chaperones in yeast has revealed an extensive interplay of inter-chaperone interactions that mediate protein homeostasis in eukaryotes [66]. Since earlier studies in *E. coli* have largely focused on reductionist biochemical analyses of single or closely related chaperones in isolation, the extent of functional connectivity between bacterial chaperones and their cofactors and substrates has not been explored systematically [67]. We address this gap by examining the global epistatic relationships of 22 general, widely conserved bacterial chaperones and ATP-dependent proteases, including ribosome-associated trigger factor (*tig*), and members of the Hsp40 (*cbpA*, *djlA*, *dnaJ*, *hscB*), Hsp70 (*dnaK*, *hscA*, *hscC*, *yegD*), Hsp90 (*htpG*), Hsp100 (*clpA, clpB, clpX, hslU*), small HSPs (*hsp33*, *ibpA*, *ibpB*), and ATP-dependent proteases (*clpP*, *ftsH*, *hslV*, *lon*) (Figure 6A, Table S1).

By applying the same strict filtering criteria (|*S*-score≥3|; *P*≤0.05) as previously, a network of 3,816 high-confidence GIs involving one or more of these factors (Table S2), revealed functional redundancy and cross talk between these determinants of protein stability. For example, a sub-network of alleviating GIs (Figure 3B) connected the ATP-dependent molecular chaperone, *clpX*, and its serine protease, *clpP*, with other known and putative chaperones/co-factors, such as the ATP-dependent protease (*hslV*), small heat shock proteins (HSPs) (*ibpA*), and *hsp100* (*clpA*), presumably reflecting functional cooperation in substrate recognition and degradation [68]–[70].

While the number of GIs identified per chaperone varied significantly, ranging from 6 (e.g., *hsp33*) to well over 600 (e.g., *cbpA*), with chaperones localized in the cytosol showing the highest connectivity (Figure 6A, Table S6), many non-chaperone genes in this sub-network interacted preferentially with a single chaperone, consistent with a specific role in protein folding (Table S7). For example, while the *dnaJ* chaperone paralog *cbpA* showed strong aggravating interactions with over 200 non-chaperones, the NAD-dependent malate dehydrogenase, *sfcA* only interacted with Hsp70 chaperone, *dnaK*. In contrast to most soluble proteins, the outer membrane porin, *ompA*, interacted with 10 different chaperones (Table S8), reflecting the multiphasic nature of membrane protein, secretion, transport, and assembly.

### Functional dependencies among chaperone systems

As each gene in the GI network possesses a GI profile, or signature, describing its functional interactions with other tested genes, the biological roles of incompletely characterized components can be inferred based on their GI profile correlation with annotated genes [6], [16], [21] (Table S9). To filter high-confidence correlations, we chose a PCC cut-off score (≥0.3) that captured roughly 18% (438 of 2,385) of the correlated gene pairs mapping to well-annotated EcoCyc complexes or pathways (Figure S3A, Protocol S12).

As implied by the GI network, the correlated GI profiles showed strong functional coordination among distinct chaperone systems (Figure 6B, Table S9). An illustrative example is the highly correlated (*r_ibpA, ibpB_*>0.5) interaction profiles of two small HSPs, *ibpA* and *ibpB*, which prevent irreversible protein aggregation due to high temperature [71], [72] (Figure 6B). Likewise, a strong correlation was observed between the ATP-dependent protein unfoldases, *clpX* and *clpA* (Figure 6B), consistent with their documented cooperation in maintaining client protein stability [73].

To gain insight into the prevalence of functional dependencies between protein complexes and chaperones, we next assessed the degree to which protein complexes were enriched with aggravating or alleviating interactions involving chaperones. We observed that roughly half of all putative soluble protein complexes showed significant (*p*-value ≤0.05) enrichment for alleviating interactions involving one or more of the 18 chaperone containing protein complexes compiled from our own large-scale proteomics survey [2] and the EcoCyc database (Figure 6C, Protocol S13). Large complexes related to general metabolism and envelope biogenesis interacted with multiple chaperones (Table S10). Chaperone-related complexes shared many non-chaperone interactors, as evidenced by high Jaccard similarity indices, suggesting functional cooperation in complex formation or maintenance (Figure 6D, Table S10, Protocol S13). Strikingly, ATP-dependent proteases, such as *clpP* interacted strongly with members of the small HSPs and Hsp100 families (Figure 6E, Table S10), consistent with previously reported interplay in protein folding and quality control [74], [75]. Likewise, GIs connected members of the Hsp100 and Hsp70 families (Figure 6E), likely reflecting Hsp100's role in rescuing protein aggregates caused by defects in Hsp70-mediated protein folding [76]. As well, members of the Hsp40 and Hsp90 systems showed strong genetic crosstalk (Figure 6E), consistent with current models of system dependencies between these chaperones [77].

### Functional modules enriched for GIs

Despite the scope of the screens, the experimentally mapped GI network of *E. coli* is sparse. To glean additional insights into the functional organization of bacterial processes, we combined our GI data with alternate evidence of functional associations, such as physical interaction information and GC-based inferences, analogous to integrative studies reported in yeast [20], [23], [78]. In particular, we examined a previously published set of 316 putative *E. coli* functional modules [2], [3], encompassing protein complexes and 43% (1,784) of all 4,145 known protein-coding genes in *E. coli* (Table S11), probing for significant enrichment of GIs between modules.

Although only ∼5% (104) of these components were screened as query mutants by eSGA, we observed significant enrichment of GIs between certain functional groupings, or modules, either as protein complexes or overlapping pathways (Figure S3B). After applying stringent permutation testing (Protocol S14), we identified 302 significant enrichments (*p*-value ≤0.05), of which the vast majority (99%) occurred between different modules (Figure S3C, Table S12). As reported for yeast [20], [22], aggravating GIs were far more prevalent than alleviating interactions between modules (Figure S3D).

The preponderance of GIs between modules provided an opportunity to explore the nature of functional crosstalk between biological systems (Figure S4A, Table S13). For example, the Suf Fe-S cluster biosynthetic module, members of the DNA polymerase module involved in proofreading and correcting replication errors via exonuclease activity, and components of the Psp (phage shock protein) system, mediating cellular responses to envelope instability and maintaining respiratory chains in *E. coli*, showed a remarkably high degree of interconnectivity (Figure S4B).

In addition to previously noted strong aggravating GIs with the functionally equivalent Isc Fe-S system (encoded by *iscRSUA-hscBA*) [13], particularly evident (Figure S4B) from the Suf module (*sufABCDSE*) were aggravating crosstalk with the vitamin B_12_ transport system, which participates in the *E. coli* response to reactive oxygen species [79]. Fe-S clusters play important roles in sensing redox/oxidative stress and iron homeostasis [80], and their breakdown can lead to accumulation of reactive oxygen species that triggers an adaptive response [81]. Structural similarity between certain components (e.g., *btuD* vs. *sufC*) [82] is also suggestive of functional dependency.

Functional coupling was also evident between the Psp (phage shock protein) and cell-envelope associated modules, such as Sap (sensitive to antimicrobial peptides), Mgl (β-methylgalactoside transporter), Mdt (multidrug resistance exporter) and Nar (Nitrate reductase) transporters, as well as with members of purine salvage pathway (Figure S4B), consistent with joint participation in respiration, maintenance of proton-motive force, and envelope integrity [83]–[86].

Conversely, alleviating interactions were preferentially detected among different module pairs, such as between the small heat shock chaperones (e.g., *ibpAB*) and multidrug efflux transporters (*acrAB*-*tolC*) (Figure S4B), possibly reflecting the active secretion of toxic protein degradation products [87]. Genes encoding members of the AAA+ family of proteases such as *clpA*-*clpP* and *hslV*-*clpP*, exhibited strong alleviating interactions with the *hslV*-*ftsH* protein quality control factors [69], suggesting they work in union (Table S12). On an individual component level, alleviating interactions often occurred between structurally similar proteins, such as the energy-dependent proteases *hslV* and *clpP* underlying a common mechanism in protein degradation [88].

### Evolutionary conservation of bacterial complexes and pathways

**Figure 7:**
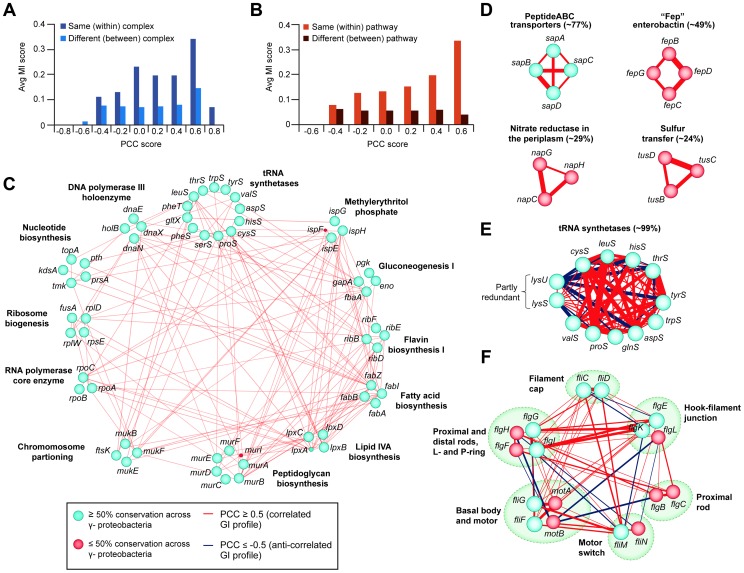
Correlated GI profiles of co-conserved genes and modules. (A) Distribution of MI and PCC score for *E. coli* gene pairs belonging to the same or different protein complexes, or (B) EcoCyc pathways. (C) Large interconnected clique of highly correlated (GI PCC score ≥0.5) and co-conserved (MI score ≥0.2 indicating high proportion of ortholog detected in γ-proteobacterial species) essential components of annotated bacterial pathways and complexes; classifications according to broad COG functional groupings. (D) Set of correlated co-conserved clusters specific to γ-proteobacteria (*sap*) or closely-related *E. coli* serotypes (*fep*, *nap*, *tus*). (E) Anti-correlated GI profiles between two partly redundant lysyl-tRNA synthetases (*lysS*, *lysU*) and other conserved tRNA determinants, and (F) between conserved components of bacterial flagellum complex. The percentage (E, F) indicates the average conservation of annotated complexes or pathways. Edge colors indicate GI profile similarity (red, correlated; dark blue, anti-correlated), edge width reflects gene-pair co-conservation (MI score), while node size or color indicates proportion of genes conserved in γ-proteobacteria or related species (blue, ≥50% conservation; red, ≤50% conservation).

Given that a large proportion of *E. coli* genes are conserved among a majority of bacteria, particularly among closely related γ-proteobacterial species [2], [10], we investigated the evolutionary significance of the putative functional associations detected by eSGA in *E. coli* by examining co-conservation of orthologs among other sequenced prokaryotes. Phylogenetic profiles were created by retrieving orthologous groups across a total of 233 fully sequenced γ-proteobacterial genomes (29 closely-related *E. coli* serotypes, 64 enterobacterial and 140 γ-proteobacterial species) from the eggNOG database [89] (Table S14). These profiles were used to derive mutual information (MI) scores based on the degree of similarity in the pattern of co-conservation of a given pair of genes (Protocol S15). We focused on gene pairs having correlated GI profiles in *E. coli* with a PCC score of ≥0.3, which favored interactions among components of the same complex and pathway (Figure S3). Consistent with biological expectation, co-conserved subunits of *E. coli* modules tended to possess highly correlated GI profiles on average compared to those belonging to different (i.e., between) complexes or pathways (Figure 7A and 7B).

Applying an MI score cut-off ≥0.2, representing a probability of co-conservation more significant than expected by random chance (Figure S5A and S5B), revealed several functionally highly correlated (*r*≥0.5) co-conserved clusters in γ-proteobacterial species (Table S15, Figure S5B). These included essential *E. coli* factors functioning in core bacterial bioprocesses such as envelope biogenesis, gluconeogenesis, and RNA/DNA/protein synthesis, which were all highly inter-connected by GIs (Figure 7C).

Furthermore, this analysis revealed varying degrees of functional correlation (i.e., at greater or less than 50% conservation) between several large, co-conserved, but non-essential bacterial protein complexes. For example, orthologues of the substrate (e.g., Sap and Fep ABC transporters) and proton (e.g., periplasmic nitrate reductase) transporter complexes, as well as the sulfur relay heterohexameric TusBCD machinery (Figure 7D), were all evolutionarily co-conserved, consistent with their broad functional importance across γ-proteobacterial species. Surprisingly, however, some subunits of highly co-conserved complexes and pathways had notable differences in their GI profiles. For example, two partly redundant, non-essential, highly conserved lysyl-tRNA synthetases of *E. coli*, *lysU* and *lysS*, each capable of sustaining protein synthesis [90], [91], were functionally anti-correlated with other tRNA synthetases (e.g., *thrS*, *tyrS*) (Figure 7E). This suggests opposing functions in support of translation, consistent with previous reports of distinct functions for these genes [90], [91]. Likewise, anti-correlated GI profiles were observed among subunits of the flagellum complex, which were largely found in closely-related *E. coli* serotypes and enterobacterial species, but which lacked orthologs among other γ-proteobacteria (Figure 7F), suggesting specialized roles in flagellum assembly [92].

Since co-conservation and correlated GI profiles reflect shared functionality [93], [94], we were able to delineate specific biological relationships. For example, the co-conserved components of the ferric enterobactin ABC transporter (e.g., *fepBCD*) and enterobactin synthetases (e.g., *entBE*) (Figure S5C) showed highly correlated GI profiles, consistent with their joint participation in iron homeostasis [95], [96]. Likewise, significant correlation was observed among the subunits of the sulfur transfer mediator (e.g., *tusBCD*) and the thiamin (e.g., *thiCDEFM*) biosynthesis machinery (Figure S5C), both of which participate in thiamin production [97], [98].

## Discussion

The vast majority (>90%) of *E. coli's* genes are dispensable for viability under standard laboratory culture conditions [19]. Unbiased interaction screens are increasingly being used to characterize the biological organization of *E. coli* [1], [2], [13], [14], [16]. Yet despite being one of the most heavily studied bacteria, nearly one-third of *E. coli*'s genes currently lack experiment-based functional annotations [1]. While proteomics and GC approaches are valuable for understanding how bacterial gene products associate into discrete biological entities (i.e., protein complexes) [2], [3], [99], they often fail to reveal higher order (i.e., pathway-level) functional relationships and process cross-talk that underlie genetic redundancy, impeding systems-level modeling [100], [101].

Genetic screens have long been appreciated as a powerful means for probing biological relationships in bacteria, but historically these studies have been focused on individual genes, complexes, or pathways in isolation [1], [6], [16]. Recent technical advances, including the development of high-throughput methods such as eSGA, GIANT-coli, and Tn-Seq [13]–[16], now permit the systematic mapping of epistatic dependencies.

In the present study, we have markedly expanded on previous initial surveys of the bacterial GI space [13], [16], achieving a scope for a prokaryote that begins to approach that reported for yeast [24], [102]. Our current GI map, although still sparse, encompasses virtually the entire *E. coli* proteome. Given the functional information contained within the recorded GI patterns, this map, despite being incomplete, represents a substantial resource for mechanistic prediction. In this study, based solely on our GI data, we were able to discover novel components and unexpected connections in well-studied pathways essential for bacterial fitness such as the association of RavA and ViaA with Fe-S and cysteine assembly, and the implication of the previously uncharacterized component YaiF in maintaining ribosomal integrity, especially in preserving translational fidelity and protein synthesis. The GI map also provides insights into the global architecture of convergent and compensatory pathway crosstalk that contributes to the overall robustness of bacterial processes. To facilitate mechanistic exploration at both levels, we report all high-confidence interactions in a dedicated open web-portal (http://ecoli.med.utoronto.ca/esga), allowing examination of both individual pair-wise gene interactions and broader connectivity among bacterial complexes and biological processes.

Integrative analyses have been documented extensively in yeast [20], [21], [45], [103], however the lack of unbiased GI data has hindered such analysis in bacteria. By combining the eSGA data from this study with previously reported *E. coli* functional modules derived by physical interaction mapping and GC [2], [3], we found unexpected relationships between certain complexes and pathways. For example, by illuminating how chaperones cooperate within a bacterial cell, we revealed unforeseen functional dependencies, suggesting an overarching surveillance network maintains protein homeostasis in bacteria.

Despite deriving meaningful biological information by expanding the scope of GI data, the current network still remains sparse, as only ∼10% (∼600 K out of ∼8 million) of all possible digenic mutant *E. coli* gene pairs were evaluated by eSGA to date. Hence, we have likely missed important patterns of connectivity that potentially biases our global inferences, leading to an underestimation of the extent of process crosstalk. However, our integrated approach revealed several novel functional associations between functional modules with significant enrichment in inter-module GIs, revealing various pathways and complexes that participate in related biological processes. This present shortfall will be overcome as the coverage of available GI data improves over the coming years and will provide a greater understanding of the functional organization of the bacterial cell.

The ability to extrapolate the epistatic connectivity diagram of *E. coli* to other microbial species lacking experimental information provides a conceptual framework for exploring bacterial evolution across different lifestyles and phylogenetically diverse microbiomes [104]. Our preliminary exploration of the co-conservation of genes and functional modules with correlated GI profiles among γ-proteobacteria illustrates the potential to outline possible adaptations, such as connectivity between iron-import and sulfonation in the biogenesis of thiamin utilization, which are linked to bacterial pathogenesis of enteric bacteria [97], [105], [106]. Thus, epistatic interactions can describe how sequence evolution in bacterial species drives functional specialization, environmental adaptations, and, potentially, speciation.

## Materials and Methods

Bacterial strains used in this study are listed in Table S16 and Protocol S16. Procedures used for the compilation of donor query targets for eSGA, strain construction, eSGA screens, computational processing epistatic interaction data to derive high confidence GI scores, the analysis of GI network properties, monochromatic analysis, computing correlation scores using GI profiles, enrichment of GI associations within and between functional modules, evolutionary conservation, phenotypic assays, as well as other relevant methods are described in detail in Supplementary Information. Network graphs were generated using Cytoscape (ver. 2.8.2), and the heat-maps were generated using in-house JAVA scripts or MATLAB.

## Supporting Information

Figure S1 Biological properties of the GI network. (A, B) The network degree distribution (A) and connectivity (B) of high-confidence aggravating (red) and alleviating (green) GIs. ACC represent average clustering coefficient. (C, D) Degree connectivity (C) and overall network betweenness centrality (zoom-in of the distribution is shown in inset) measure (D) is shown for essential and non-essential *E. coli* genes.
(PDF)

Figure S2 Benchmarking the GI networks. (A, B, C) Distribution of correlation coefficients between GI profiles for gene pairs predicted by genomic context (GC) methods (A), co-expression (B), and phenomic [i.e., chemical-genetic interaction (CGI)] profiles (C) versus randomly drawn gene pairs. The significance value was computed using the two-sample Kolmogorov-Smirnov test. (D) Distribution of correlation coefficients between GI profiles for the last and the first gene versus the first and the middle gene in an operon.
(PDF)

Figure S3 Analyses on inter and intra-module GI pairs. (A) Precision and recall analysis on well-annotated Ecocyc complexes or pathways to determine the optimal correlation cut-off score to filter highly-correlated gene pairs than by random chance. (B) Intra- and inter-module epistatic associations among genes participating in the same protein complex or overlapping pathway. (C) Z-score distribution of genetically interacting functional module pairs [2], [3]. The corresponding Z-score for the number of interactions occurring within (i.e., intra-module GIs) or between (inter-module GIs) functional modules was calculated via permutation testing (Protocol S14). The numbers above each bar indicate the number of module pairs found within the given Z-Score bin. The red colored bars on the upper tail indicate the Z-score threshold for significantly interacting intra- and inter-module pairs, as defined by the permutation test derived from *p*-value ≤0.05. (D) Fraction of GIs enriched for aggravating or alleviating within (intra) and between (inter) modules. The denominator in each bar represents the total number of GIs tested in intra- or inter-module pairs, whereas the numerator indicate the significant GIs that are enriched (Z-Score≥2.5) in intra- or inter-module pairs. The significance value is computed using Fisher's exact test.
(PDF)

Figure S4 An integrated biological network of *E. coli* functional modules revealing novel functional links among diverse bioprocesses. (A) An overview of modules defined in our previous studies [2], [3], where each node represents a distinct cluster of *E. coli* genes sharing functional similarity, with edges representing genetic interactions (GIs) generated by our study found to be statistically enriched (Z-score≥2.5 and inter-module interactions |≥3|; see Supplemental Methods) between module pairs. Numbered circles highlight sub-networks of interest (right), describing a common biological role known to be possessed by genes composing the interacting modules. Node color indicates functional module membership in known pathways (red) or complexes (blue); edge thickness reflects number of GIs observed. The highlighted edges (white) correspond to the inter-module GIs of the indicated sub-networks shown on the periphery. (B) Statistically enriched inter-module GIs occurring between genes known to participate in various bioprocesses. Node color represents functional module membership of individual genes; edge color indicates predominant GI type (aggravating, red; alleviating, green), while node shape indicates status as query (hexagon) or recipient (circle) strains during eSGA screening.
(PDF)

Figure S5 Evolutionary conservation analyses on the bacterial complexes or pathways. (A) Precision and recall analysis to determine optimal mutual information (MI) score cut-off to identify highly conserved clusters than by random chance. (B) Relative ratio of highly conserved gene pairs in the same pathway against pairs of genes in different pathway with varying mutual information or correlated GI profile cut-offs. (C) Heat-map representing the co-conserved PCC scores among the EcoCyc complexes. Numbers indicate the PCC score that were computed using the GI profiles between recipient gene pairs. NAs indicate interactions whose MI scores could not be calculated; heat-map coloring from yellow-to-red indicates increasing correlation of GI profiles. Cells without color fall below the co-conservation threshold of MI≥0.2.
(PDF)

Protocol S1 Selection of non-redundant donor ‘query’ targets for eSGA screen.
(PDF)

Protocol S2 Processing epistatic interaction data set to derive high-confidence and significant GI scores.
(PDF)

Protocol S3 Analysis on the data quality and network properties of GI dataset.
(PDF)

Protocol S4 Compilation of interaction datasets for assessing GI data quality and enrichment analysis.
(PDF)

Protocol S5 The polarity effect of Hfr query mutant strain on the downstream genes.
(PDF)

Protocol S6 Monochromatic analysis.
(PDF)

Protocol S7 Growth curve and drug sensitivity phenotypic assays.
(PDF)

Protocol S8 Effect of Isc pathway overexpression and aminoglycoside treatment on *E. coli* growth.
(PDF)

Protocol S9 Effect of *E. coli* growth inhibition of RavA overexpression on inorganic and organic sources of sulphur.
(PDF)

Protocol S10 Immunoprecipitation.
(PDF)

Protocol S11 Ribosome profiles, translation fidelity, and cellular RNA analyses.
(PDF)

Protocol S12 Computing correlation and determining optimal cut-off score for deriving GI profiles.
(PDF)

Protocol S13 Analysis on chaperone complexes and their association with epistatic interactions.
(PDF)

Protocol S14 eSGA inter-module permutation test.
(PDF)

Protocol S15 Generation of phylogenetic co-occurrence using correlated GI profiles and MI scores.
(PDF)

Protocol S16 Bacterial strains, plasmids, and genetic screens.
(PDF)

Table S1 Catalog of donor query strains targeted in this study.
(XLS)

Table S2 List of gene pairs with high-confidence epistatic interactions.
(XLS)

Table S3 Comparison of GIs to manually curated gene pairs compiled from low-throughput experimental studies.
(XLS)

Table S4 Monochromatic GIs that are significant (sheet 1) and non-significant (sheet 2) between bacterial processes.
(XLS)

Table S5 Conservation of YaiF among microbes.
(XLS)

Table S6 Average number of GIs identified per donor query chaperone.
(XLS)

Table S7 Number of epistatic interactions between chaperone to chaperone and chaperone to non-chaperone.
(XLS)

Table S8 Epistatic interaction of non-chaperone gene to chaperones targeted in the study.
(XLS)

Table S9 Correlation coefficient scores for each gene across all other genes in the *E. coli* genome.
(XLSX)

Table S10 Number of GIs of each chaperone mutant with one or more chaperone-containing protein complexes (sheet 1-I). Presence or absence of epistatic association of a chaperone gene deletion mutant with one or more chaperone-containing protein complexes (sheet 1-II). Overlap of GIs among the chaperone-containing protein complexes (sheet 2). Functional cross-talks between different chaperone families (sheet 3). List of complexes compiled from EcoCyc (sheet 4) and Hu et al (sheet 5) study.
(XLS)

Table S11 List of functional modules used in this study.
(XLS)

Table S12 Number of intra- and inter-module pairs with significantly enriched epistatic interactions (sheet 1). Genes involved in the significantly enriched intra- and inter-module pairs (sheet 2).
(XLS)

Table S13 Functional crosstalk between significantly enriched biological processes highlighted (sheet 1) and the rest (sheet 2) in the overview network.
(XLS)

Table S14 List of fully sequenced γ-proteobacterial species (sheet 1) and the percentage identity BlastN similarity of *E. coli* K-12 W3110 (the lab *E. coli* strain included in eggNOG) against the 233 γ-proteobacterial species used to generate the phylogenetic profiles for calculating MI scores (sheet 2). Phylogenetic profiles of eSGA recipient genes across 233 γ-proteobacterial species (sheet 3). Proportion of recipient genes conserved across closely-related *E. coli* serotypes, enterobacterial, and γ-proteobacterial species (sheet 4).
(XLS)

Table S15 Identification of several distinct, highly correlated clusters of bioprocesses with varying patterns of co-conservation.
(XLSX)

Table S16 List of bacterial strains and plasmids used in this study.
(XLS)
