# Supplementary figures and images for: Quantitative Genome-Wide Genetic Interaction Screens Reveal Global Epistatic Relationships of Protein Complexes in Escherichia coli
Source: PLoS Genet. 2014 Feb 20;10(2):e1004120. doi: 10.1371/journal.pgen.1004120 (PMC3930520; doi:10.1371/journal.pgen.1004120)

A

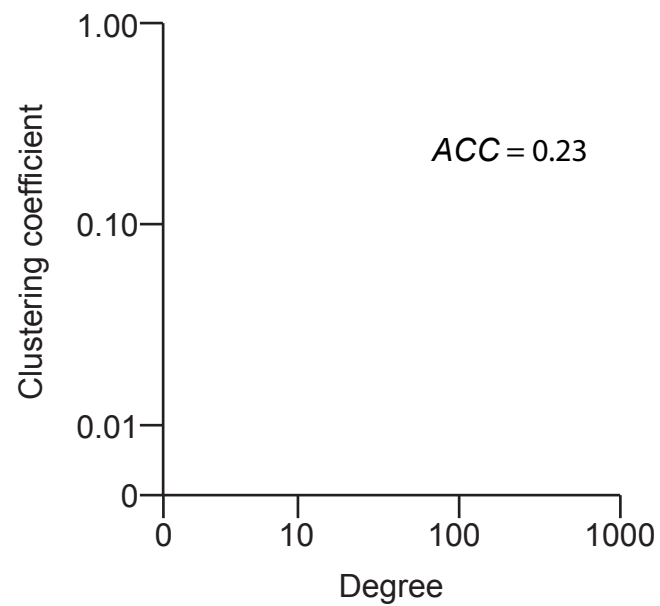

B

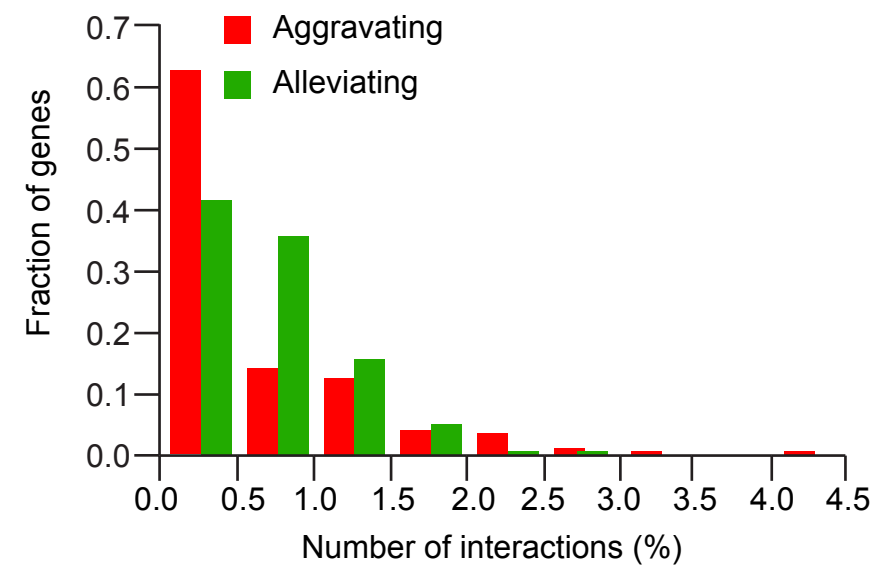

C

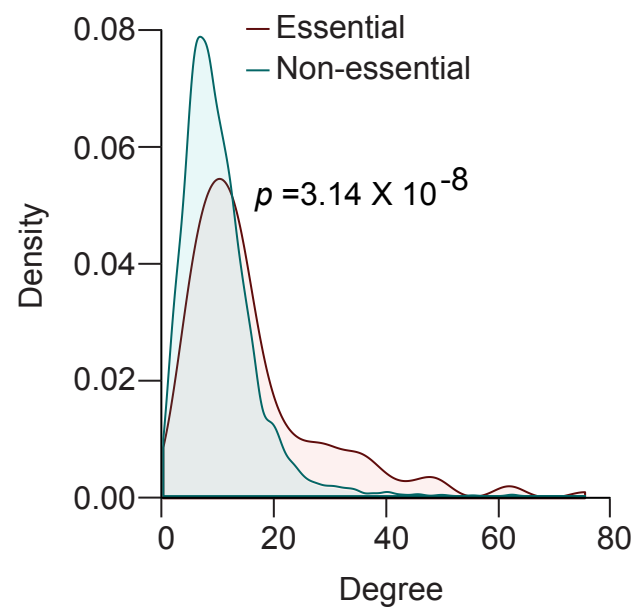

D

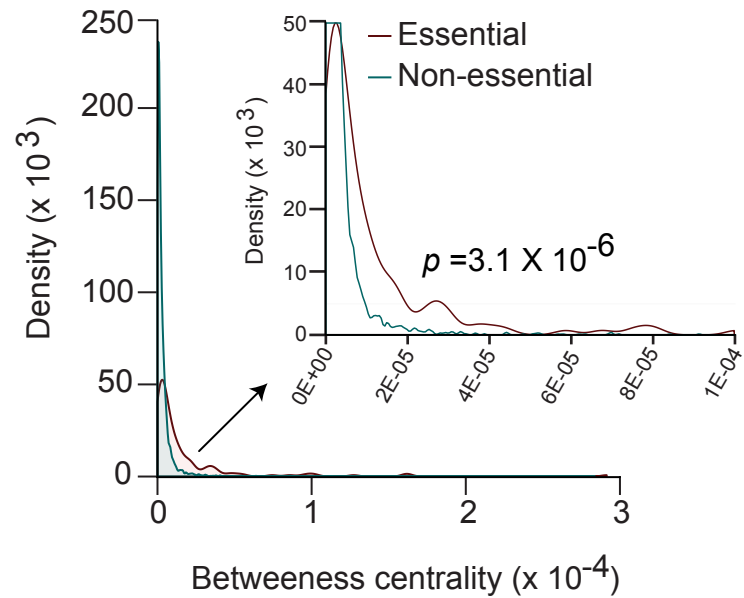

Figure S1

Supplement: Figure S1 — Biological properties of the GI network. (A, B) The network degree distribution (A) and connectivity (B) of high-confidence aggravating (red) and alleviating (green) GIs. ACC represent average clustering coefficient. (C, D) Degree connectivity (C) and overall network betweenness centrality (zoom-in of the distribution is shown in inset) measure (D) is shown for essential and non-essential E. coli genes. (PDF) [file pgen.1004120.s001.pdf]

**A**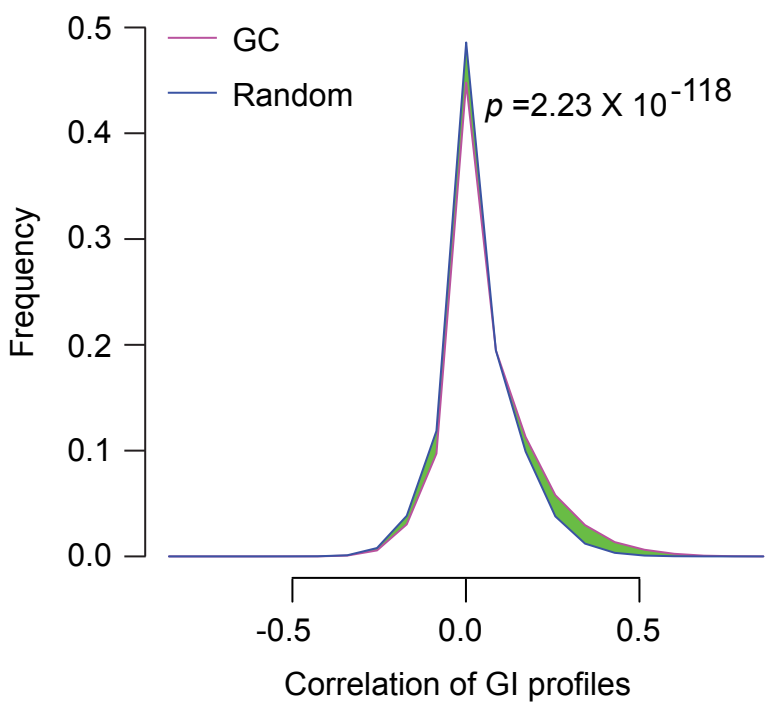**C**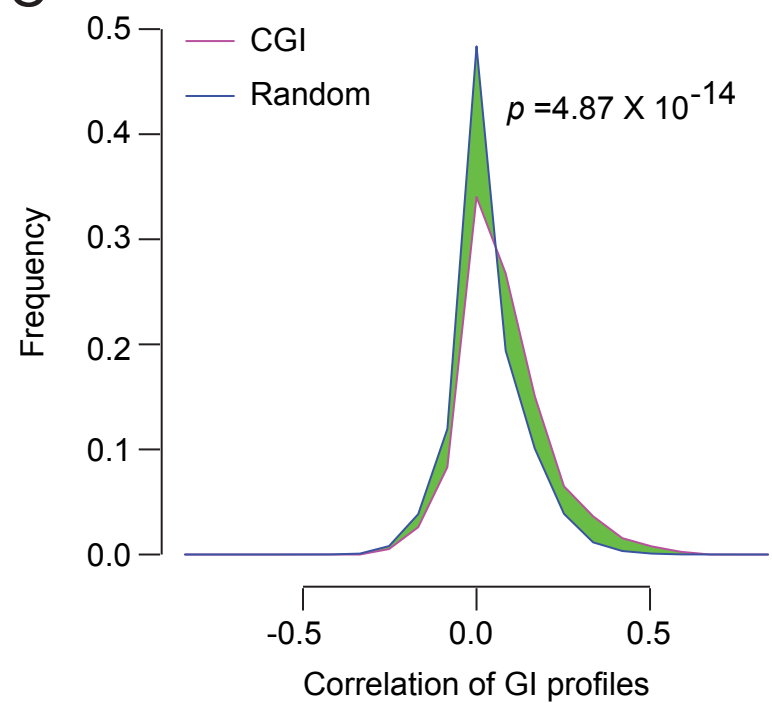**B**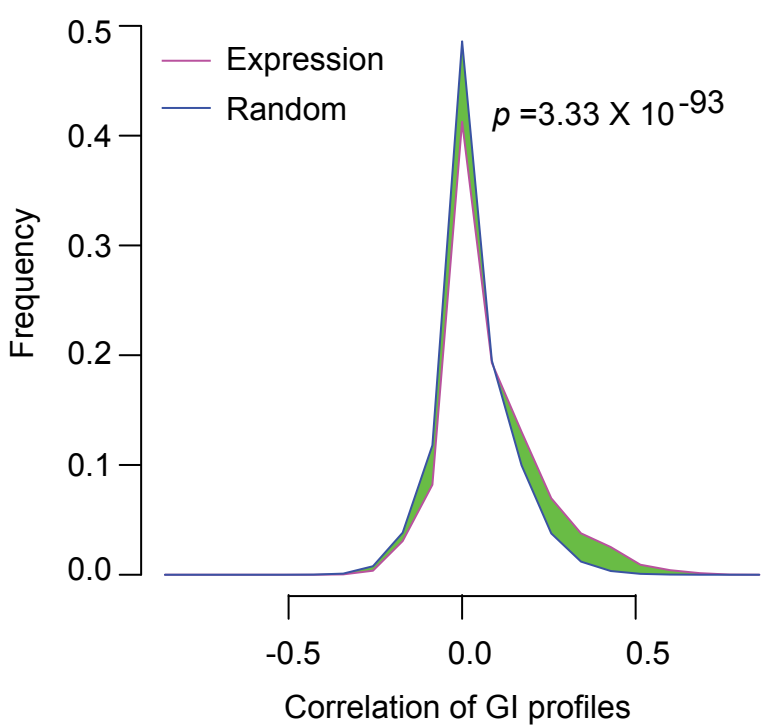**D**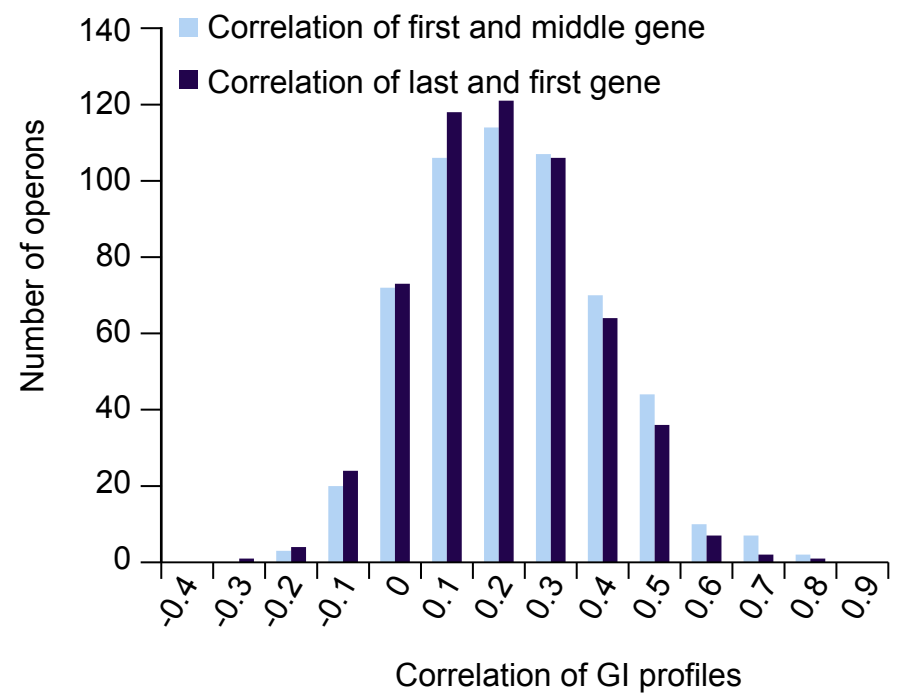**Figure S2**

Supplement: Figure S2 — Benchmarking the GI networks. (A, B, C) Distribution of correlation coefficients between GI profiles for gene pairs predicted by genomic context (GC) methods (A), co-expression (B), and phenomic [i.e., chemical-genetic interaction (CGI)] profiles (C) versus randomly drawn gene pairs. The significance value was computed using the two-sample Kolmogorov-Smirnov test. (D) Distribution of correlation coefficients between GI profiles for the last and the first gene versus the first and the middle gene in an operon. (PDF) [file pgen.1004120.s002.pdf]

A

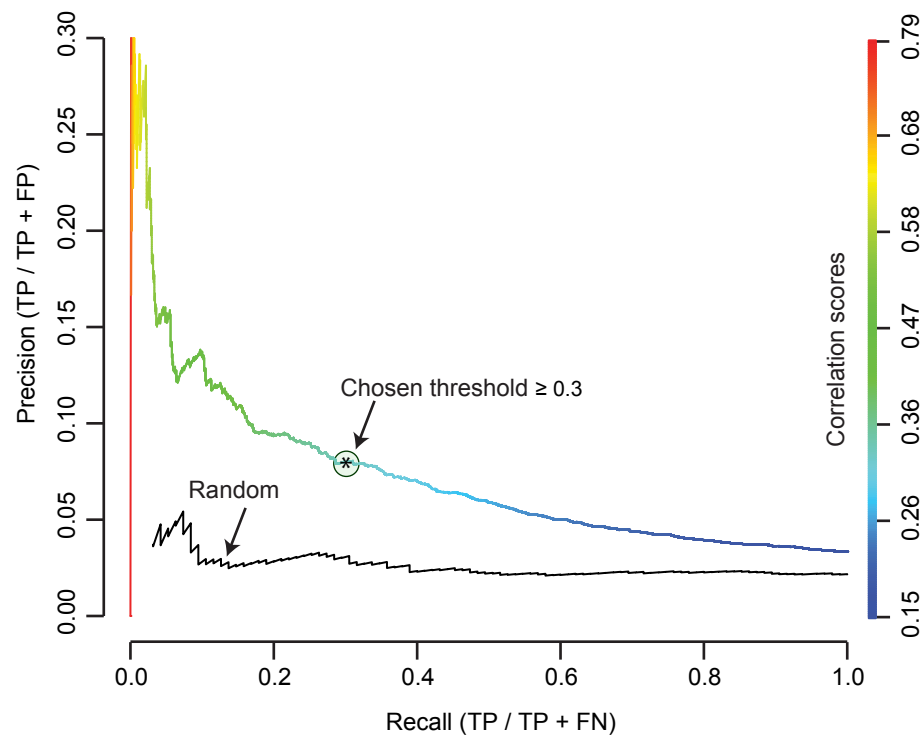

B

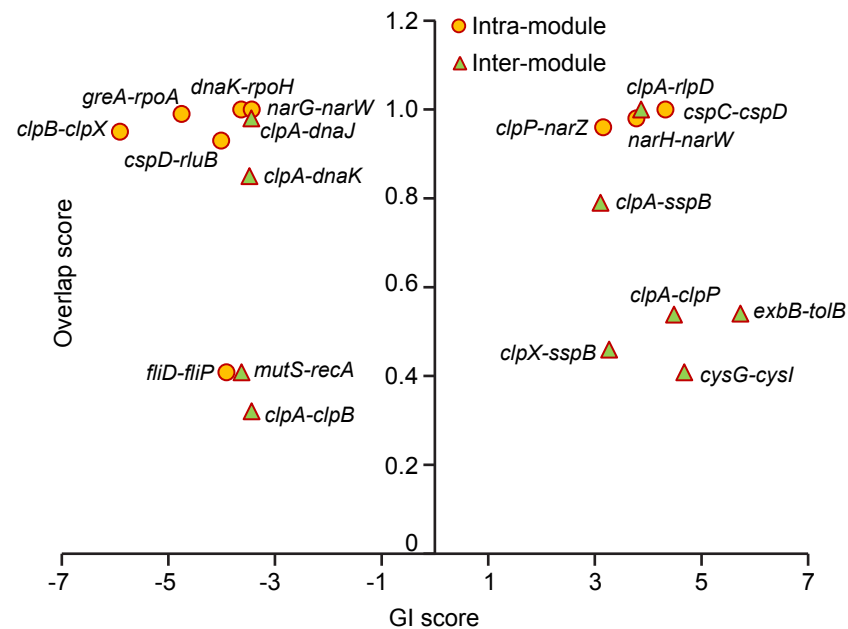

C

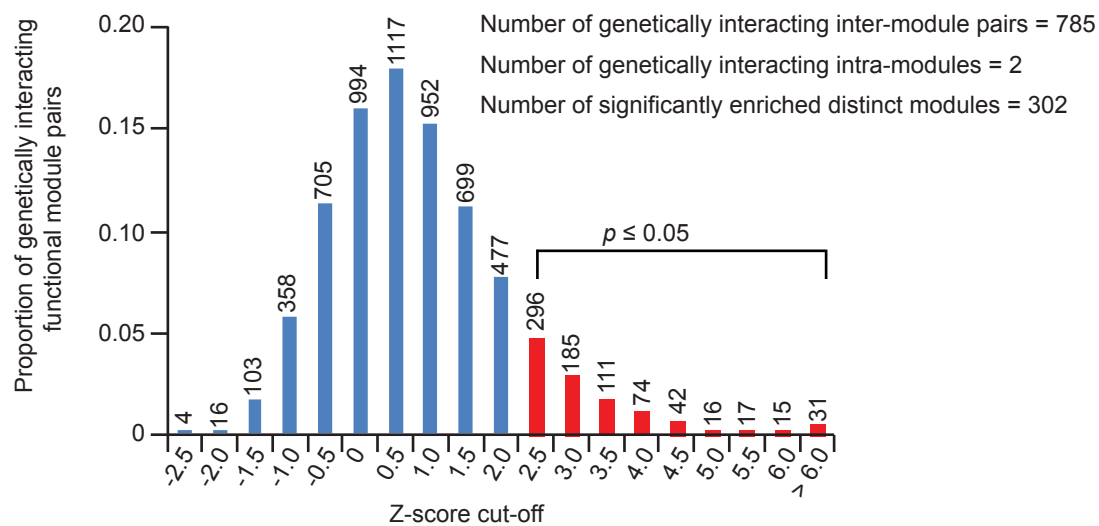

D

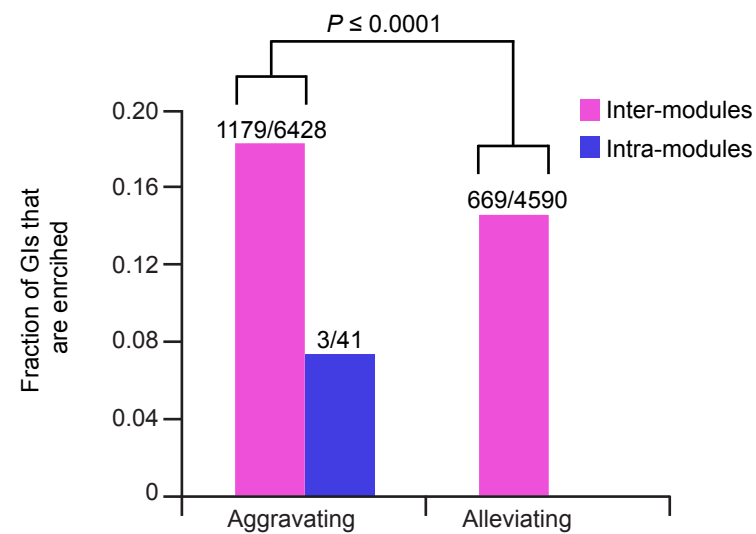

Figure S3

Supplement: Figure S3 — Analyses on inter and intra-module GI pairs. (A) Precision and recall analysis on well-annotated Ecocyc complexes or pathways to determine the optimal correlation cut-off score to filter highly-correlated gene pairs than by random chance. (B) Intra- and inter-module epistatic associations among genes participating in the same protein complex or overlapping pathway. (C) Z-score distribution of genetically interacting functional module pairs [2], [3]. The corresponding Z-score for the number of interactions occurring within (i.e., intra-module GIs) or between (inter-module GIs) functional modules was calculated via permutation testing (Protocol S14). The numbers above each bar indicate the number of module pairs found within the given Z-Score bin. The red colored bars on the upper tail indicate the Z-score threshold for significantly interacting intra- and inter-module pairs, as defined by the permutation test derived from p-value ≤0.05. (D) Fraction of GIs enriched for aggravating or alleviating within (intra) and between (inter) modules. The denominator in each bar represents the total number of GIs tested in intra- or inter-module pairs, whereas the numerator indicate the significant GIs that are enriched (Z-Score≥2.5) in intra- or inter-module pairs. The significance value is computed using Fisher's exact test. (PDF) [file pgen.1004120.s003.pdf]

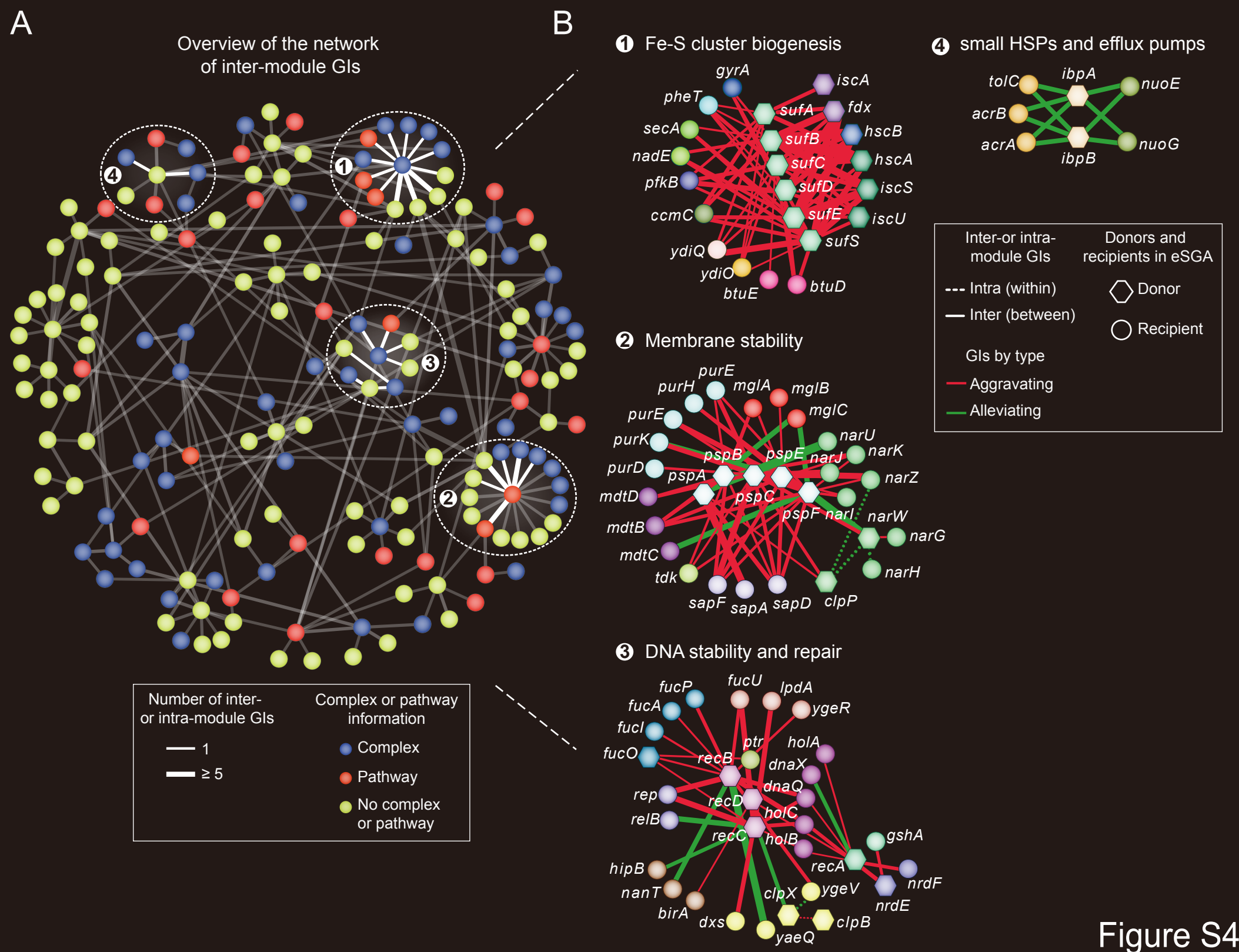

Figure S4

Supplement: Figure S4 — An integrated biological network of E. coli functional modules revealing novel functional links among diverse bioprocesses. (A) An overview of modules defined in our previous studies [2], [3], where each node represents a distinct cluster of E. coli genes sharing functional similarity, with edges representing genetic interactions (GIs) generated by our study found to be statistically enriched (Z-score≥2.5 and inter-module interactions |≥3|; see Supplemental Methods) between module pairs. Numbered circles highlight sub-networks of interest (right), describing a common biological role known to be possessed by genes composing the interacting modules. Node color indicates functional module membership in known pathways (red) or complexes (blue); edge thickness reflects number of GIs observed. The highlighted edges (white) correspond to the inter-module GIs of the indicated sub-networks shown on the periphery. (B) Statistically enriched inter-module GIs occurring between genes known to participate in various bioprocesses. Node color represents functional module membership of individual genes; edge color indicates predominant GI type (aggravating, red; alleviating, green), while node shape indicates status as query (hexagon) or recipient (circle) strains during eSGA screening. (PDF) [file pgen.1004120.s004.pdf]
