# Supplementary material for: Quantitative Genome-Wide Genetic Interaction Screens Reveal Global Epistatic Relationships of Protein Complexes in Escherichia coli
Source: PLoS Genet. 2014 Feb 20;10(2):e1004120. doi: 10.1371/journal.pgen.1004120 (PMC3930520; doi:10.1371/journal.pgen.1004120)
Supplement: Figure S5 — Evolutionary conservation analyses on the bacterial complexes or pathways. (A) Precision and recall analysis to determine optimal mutual information (MI) score cut-off to identify highly conserved clusters than by random chance. (B) Relative ratio of highly conserved gene pairs in the same pathway against pairs of genes in different pathway with varying mutual information or correlated GI profile cut-offs. (C) Heat-map representing the co-conserved PCC scores among the EcoCyc complexes. Numbers indicate the PCC score that were computed using the GI profiles between recipient gene pairs. NAs indicate interactions whose MI scores could not be calculated; heat-map coloring from yellow-to-red indicates increasing correlation of GI profiles. Cells without color fall below the co-conservation threshold of MI≥0.2. (PDF) [file pgen.1004120.s005.pdf]

A

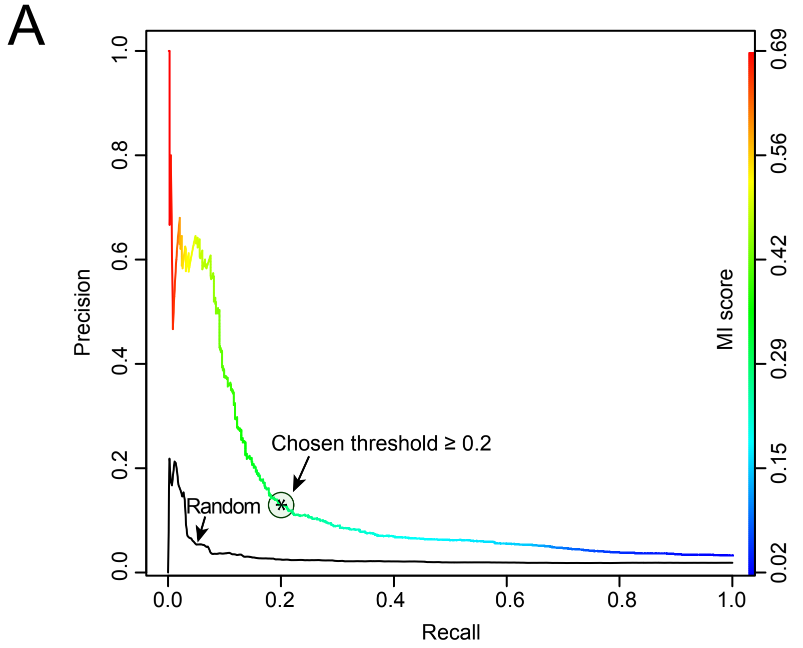

B

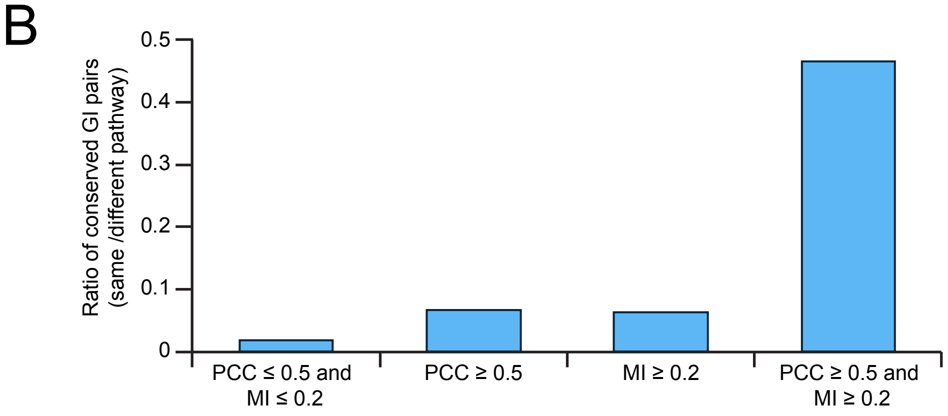

C

Complexes	Genes	"Fep" enterobactin ABC transporter		
		fepC	*fepB*	*fepD*
Enterobactin synthetases	*entE*	0.62	0.49	0.51
	entB	0.58	0.39	0.35

Genetic profile similarity (PCC scores)
Most correlated
Least correlated

Complexes	Genes	"Fep" enterobactin ABC transporter			Enterobactin synthetases	
		fepC	*fepB*	*fepD*	*entE*	*entB*
Sulfur transfer	*tusB*	0.20	0.17	0.19	0.16	0.16
	tusC	0.33	0.40	0.30	0.36	0.30
	tusD	0.38	0.45	0.39	0.45	0.38

Complexes	Genes	"Fep" enterobactin ABC transporter			Enterobactin synthetases	
		fepC	*fepB*	*fepD*	*entE*	*entB*
Thiamin biosynthesis I	*thiE*	0.46	0.33	0.30	0.34	0.44
	thiC	0.20	0.15	0.05	0.15	0.23
	thiF	0.09	NA	0.06	0.15	0.20
	thiM	0.07	0.21	0.17	0.04	0.05
	thiD	0.16	0.35	0.13	0.04	0.16

Complexes	Genes	"Fep" enterobactin ABC transporter			Enterobactin synthetases		Sulfur transfer		
		fepC	*fepB*	*fepD*	*entE*	*entB*	*tusB*	*tusC*	*tusD*
Xanthine dehydrogenase	*xdhA*	0.30	0.32	0.32	0.14	NA	0.53	0.50	0.52
	xdhB	NA	0.03	NA	0.33	0.23	0.19	0.27	0.23
	xdhC	0.18	0.16	0.13	0.21	0.14	0.27	0.16	0.32
Glycerol-3-phosphate-dehydrogenase	*glpA*	0.39	0.44	0.37	0.38	0.29	0.11	0.38	0.38
	glpB	0.48	0.38	0.40	0.35	0.47	0.05	0.26	0.31
	glpC	NA	0.02	NA	NA	NA	0.02	NA	NA

Figure S5
