# Supplementary material for: Quantitative Genome-Wide Genetic Interaction Screens Reveal Global Epistatic Relationships of Protein Complexes in Escherichia coli
Source: PLoS Genet. 2014 Feb 20;10(2):e1004120. doi: 10.1371/journal.pgen.1004120 (PMC3930520; doi:10.1371/journal.pgen.1004120)
Supplement: Protocol S1 — Selection of non-redundant donor ‘query’ targets for eSGA screen. (PDF) [file pgen.1004120.s006.pdf]

Protocol S1. Selection of non-redundant donor ‘query’ targets for eSGA screen

The list of *E. coli* donor ‘query’ genes targeted for eSGA screens (Table S1) was based on large-scale proteomics data generated in house [1,2], where the PPI degree was computed for a given bait or prey protein against all other proteins in *E. coli*, and genes were chosen on the basis of their products having several predicted interactions (high degree), as long as they did not have a direct interaction with each other. Because one-third of the coding genes of *E. coli* are not annotated [2] we made sure that the proteins encoded by any chosen gene had a minimal evidence of being *bona fide*. Briefly, four different proteomic approaches were used in the identification of *E. coli* proteins, which includes spectral generation from an LTQ tandem mass spectrometer using the *E. coli* DY330 whole cell lysate; and (ii-iv) from the affinity purified cytosolic *E. coli* proteins using the LC-MS/MS (LCQ and LTQ) and peptide mass fingerprinting (MALDI-TOF) mass spectrometers. The identified proteins were then ranked based on the number of experiments showing their presence. For instance, proteins detected in all four different methods were given a ranking of 1, while those detected in three, two, and one were ranked as 2, 3, and 4, respectively. From each of these rankings, we chose a selected subset of non-redundant target query genes that are as functionally divergent as possible not only based on their interaction connectivity but also on their critical role in *E. coli* physiology.

References:

1. Butland G, Peregrín-Alvarez JM, Li J, Yang W, Yang X, et al. (2005) Interaction network containing conserved and essential protein complexes in Escherichia coli. Nature 433: 531-537.
2. Hu P, Janga SC, Babu M, Diaz-Mejia JJ, Butland G (2009) Global functional atlas of Escherichia coli encompassing previously uncharacterized proteins. PLoS Biol 7: e1000096.
