# Supplementary material for: Quantitative Genome-Wide Genetic Interaction Screens Reveal Global Epistatic Relationships of Protein Complexes in Escherichia coli
Source: PLoS Genet. 2014 Feb 20;10(2):e1004120. doi: 10.1371/journal.pgen.1004120 (PMC3930520; doi:10.1371/journal.pgen.1004120)
Supplement: Protocol S2 — Processing epistatic interaction data set to derive high-confidence and significant GI scores. (PDF) [file pgen.1004120.s007.pdf]

Protocol S2. Processing epistatic interaction data set to derive high-confidence and significant GI scores

The genome-wide eSGA genetic screen was carried out essentially as previously reported [1]. The raw colony size fitness measurements from the newly performed 124 eSGA genetic screens were normalized and processed into *S*-scores essentially as described in our previous genome-wide study [1]. Each genome-wide screen was performed twice by replica pinning the conjugants arrayed in a 384 density format using four biological replicate recipient colonies, which is selected further on double antibiotics at 1,536 colony density, to generate double mutant colonies. These eight replicate measurements of each gene pair were subsequently averaged into a single GI *S*-score to account for colony plate variance. This newly derived GI score was then combined with our previously published GI datasets from 39 genome-wide screens to generate a comprehensive GI network. We also subsequently removed all gene pairs with a chromosomal distance of 10 kb on either side of the donor query loci to account for linkage effects that are known to cause false positives [1]. The filtered GI interaction data was *Z*-score normalized and thresholded at a cut-off value of |2|, corresponding to a p-value ≤ 0.05 and a GI *S*-score of ≤ -3.3 for aggravating and ≥ 3.1 for alleviating interactions.

References:

1. Butland G, Babu M, Díaz-Mejía JJ, Bohdana F, Phanse S, et al. (2008) eSGA: E. coli synthetic genetic array analysis. Nat Methods 5: 789-795.
