# Supplementary material for: Quantitative Genome-Wide Genetic Interaction Screens Reveal Global Epistatic Relationships of Protein Complexes in Escherichia coli
Source: PLoS Genet. 2014 Feb 20;10(2):e1004120. doi: 10.1371/journal.pgen.1004120 (PMC3930520; doi:10.1371/journal.pgen.1004120)
Supplement: Protocol S3 — Analysis on the data quality and network properties of GI dataset. (PDF) [file pgen.1004120.s008.pdf]

Protocol S3. Analysis on the data quality and network properties of GI dataset

The network betweenness for essential and non-essential genes was computed using the networkX package [1] on the unweighted GI network, which is derived by deducing an edge between two genes if the corresponding pair had a Z-score $|\geq 2|$. The significance value for essential genes in comparison to non-essential genes for betweenness and GI degree distributions were calculated using the one-sided unpaired Student's *t*-test. Furthermore, the significance value for the aggravating to alleviating ratio between essential and non-essential complexes was calculated using the Mann-Whitney-Wilcoxon Test in R.

The quality of our GI network was assessed by comparing the results against a comprehensive list of manually curated GIs from the literature. The significance of these interactions was computed using an empirical P-value by generating 10,000 randomized literature networks essentially as previously described [2]. Since the literature curated interactions do not have a GI score, we considered GI pairs as true positives as long as there was agreement with the literature curated interactions, especially in terms of their directionality (i.e., aggravating or alleviating), and the GI were above a relaxed empirical threshold cut-off *S*-score of $|\geq 2.0|$.

References:

1. Hagberg A, Swart P, Chult DS. Exploring network structure, dynamics, and function using NetworkX. In: Varoquaux G, Vaught T, Millman J, editors; 2008; Pasadena, CA, USA. pp. 11--15.
2. Babu M, Díaz-Mejía JJ, Vlasblom J, Gagarinova A, Phanse S, et al. (2011) Genetic interaction maps in Escherichia coli reveal functional crosstalk among cell envelope biogenesis pathways. PLoS Genet 7: e1002377.
