# Supplementary material for: Quantitative Genome-Wide Genetic Interaction Screens Reveal Global Epistatic Relationships of Protein Complexes in Escherichia coli
Source: PLoS Genet. 2014 Feb 20;10(2):e1004120. doi: 10.1371/journal.pgen.1004120 (PMC3930520; doi:10.1371/journal.pgen.1004120)
Supplement: Protocol S4 — Compilation of interaction datasets for assessing GI data quality and enrichment analysis. (PDF) [file pgen.1004120.s009.pdf]

Protocol S4. Compilation of interaction datasets for assessing GI data quality and enrichment analysis

To evaluate the GI data quality, we extracted the following data sets: (i) protein complexes and high-quality reference *E. coli* protein-protein interaction (PPI) data sets were downloaded from EcoCyc (downloaded as of December 12, 2012) and eNET [1] databases; (ii) co-expressed genes from microarray based mRNA transcript profiles were downloaded from the M3D database [2] and were compiled essentially as previously described [3]; (iii) chemical phenotypic data was obtained from a recent study [4], where a library of *E. coli* strains either deleted for non-essential components or hypomorphic alleles of essential genes were screened against hundreds of conditions to study gene essentiality; and (iv) the literature curated gene pairs used in the comparison of genome-wide GI network is essentially a manual compilation from several low throughput experimental studies [3,5]. The functional modules inferred from GC and PPI methods were obtained from our previous computational and large-scale experimental studies [1,6] to assess significant enrichment of GI associations within and between modules.

The uncentered Pearson correlation coefficient histograms calculated for pairs of GI profiles were determined for 6,132 gene pairs coding for the interacting proteins in the reference *E. coli* PPI set, 3,193 pairs that belonging to a common operon, 24,367 pairs for GC (i.e., at a confidence cut-off of 0.9), 385 GI pairs corresponding to mutant fitness phenotypes that were screened against hundreds of chemical assaults, and finally 6,258 pairs with an expression signal [i.e., measured in terms of RMA (Robust Multi Array values)] that were detected in standard laboratory rich media growth conditions. The random distribution was computed by averaging 100 histograms from randomly drawn gene pairs essentially as previously described [3].

Significance values for all the GI profile histograms were calculated using the Kolmogorov-Smirnov test.

References:

1. Hu P, Janga SC, Babu M, Diaz-Mejia JJ, Butland G (2009) Global functional atlas of Escherichia coli encompassing previously uncharacterized proteins. PLoS Biol 7: e1000096.
2. Faith JJ, Driscoll ME, Fusaro VA, Cosgrove EJ, Hayete B, et al. (2008) Many Microbe Microarrays Database: uniformly normalized Affymetrix compendia with structured experimental metadata. Nucleic Acids Res 36: D866-870.
3. Babu M, Díaz-Mejía JJ, Vlasblom J, Gagarinova A, Phanse S, et al. (2011) Genetic interaction maps in Escherichia coli reveal functional crosstalk among cell envelope biogenesis pathways. PLoS Genet 7: e1002377.
4. Nichols RJ, Sen S, Choo YJ, Beltrao P, Zietek M, et al. (2011) Phenotypic landscape of a bacterial cell. Cell 144: 143-156.
5. Babu M, Musso G, Diaz-Mejia JJ, Butland G, Greenblatt JF, et al. (2009) Systems-level approaches for identifying and analyzing genetic interaction networks in Escherichia coli and extensions to other prokaryotes. Mol Biosyst 12: 1439-1455.
6. Peregrin-Alvarez JM, Xiong X, Su C, Parkinson J (2009) The Modular Organization of Protein Interactions in Escherichia coli. PLoS Comput Biol 5: e1000523.
