# Supplementary material for: Quantitative Genome-Wide Genetic Interaction Screens Reveal Global Epistatic Relationships of Protein Complexes in Escherichia coli
Source: PLoS Genet. 2014 Feb 20;10(2):e1004120. doi: 10.1371/journal.pgen.1004120 (PMC3930520; doi:10.1371/journal.pgen.1004120)
Supplement: Protocol S5 — The polarity effect of Hfr query mutant strain on the downstream genes. (PDF) [file pgen.1004120.s010.pdf]

Protocol S5. The polarity effect of Hfr query mutant strain on the downstream genes

If we assume a deletion of an Hfr query strain affects the downstream genes within the same operon, then deletion of adjacent genes should usually display similar GI profiles (or phenotypes). However, that wasn't the case. For example, as we have shown previously in our original methods publication [1], when a *sufC* donor query mutant is conjugated against recipient single gene deletion mutant strains covering the entire Isc operon, synthetic sick or lethal phenotypes were seen for all the genes in Isc operon except for *iscA* and *iscR*, which are internal and terminal genes in the operon, excluding the possibility of polarity artifacts.

Moreover, we expect genes in operons to correlate, since functionally related-question: does the first gene in an operon typically show the lowest/weakest (hopefully not) GI or synthetic sick lethal scores in the screens compared to mutants lacking middle/end genes. Notably, we found that for nearly 30% of the operons (216 of 720) examined, the last and the first gene in a given operon are just as likely to be positively correlated (PCC $\geq$ 0.3) as the first and middle genes (Figure S2D); however the last gene cannot possibly underlie the GI phenotypes (and hence polarity effects) for every operon.

References:

1. Butland G, Babu M, Díaz-Mejía JJ, Bohdana F, Phanse S, et al. (2008) eSGA: E. coli synthetic genetic array analysis. Nat Methods 5: 789-795.
