# Supplementary material for: Quantitative Genome-Wide Genetic Interaction Screens Reveal Global Epistatic Relationships of Protein Complexes in Escherichia coli
Source: PLoS Genet. 2014 Feb 20;10(2):e1004120. doi: 10.1371/journal.pgen.1004120 (PMC3930520; doi:10.1371/journal.pgen.1004120)
Supplement: Protocol S6 — Monochromatic analysis. (PDF) [file pgen.1004120.s011.pdf]

Protocol S6. Monochromatic analysis

The monochromatic analysis on the high-confidence GIs was performed essentially as described [1]. Briefly, a monochromatic score was computed for each pairwise process using the ratio of aggravating to alleviating GIs based on their overall distribution in the high confidence GI network. The following equation was used to compute the monochromatic score: $\frac{1}{n}\sum xn$, where *n* is the total number of interactions observed between processes, and *xn* = 1 for alleviating and -1 for aggravating. To identify the monochromatic GIs between processes, we used MultiFun schema to group the recipient genes into 321 distinct categories, including a functional grouping for 'unknown' genes, whereas the donor query genes were grouped into 11 core bacterial processes as defined in Table S1. In total, we tested 2,693 process pairs that are associated with epistatic connections between the donor and recipient functional groups. We then employed Fishers two-sided exact test (p-value ≤ 0.05) to delineate the genuine monochromatic GIs between processes. Of the 2,693 tested pairs, we found that 125 non-redundant MultiFun processes based on the recipient genes tended to exhibit significant (p-value ≤ 0.05) monochromatic relationships with 11 donor core bioprocesses.

References:

1. Michaut M, Baryshnikova A, Costanzo M, Myers CL, Andrews BJ, et al. (2011) Protein complexes are central in the yeast genetic landscape. PLoS Comput Biol 7: e1001092.
