# Supplementary material for: Quantitative Genome-Wide Genetic Interaction Screens Reveal Global Epistatic Relationships of Protein Complexes in Escherichia coli
Source: PLoS Genet. 2014 Feb 20;10(2):e1004120. doi: 10.1371/journal.pgen.1004120 (PMC3930520; doi:10.1371/journal.pgen.1004120)
Supplement: Protocol S7 — Growth curve and drug sensitivity phenotypic assays. (PDF) [file pgen.1004120.s012.pdf]

Protocol S7. Growth curve and drug sensitivity phenotypic assays

Overnight cultures of deletion mutants and parental strains, grown at 32 °C in LB rich medium were inoculated into a 100-well microtitre plate containing 100 µl of LB medium, as required. The cells were subjected to constant shaking at 32 °C, and the optical densities of the culture were determined at 600 nm (OD_{600}) using a Bioscreen C automated microbiology growth curve analysis system (Thermolabsystems, Helsinki, Finland) every 15 min for until 24 hrs. In the case of *cysB* mutant fitness experiment, 200-µl aliquots of the mutant and wild type cultures in a standard 96-well plate was monitored via OD_{600} measurements at designated time points. The absorbance was quantified using the EnSpire 2300 Multilabel Reader (Perkin Elmer).

For the drug assay, overnight cultures in LB medium were serially diluted and pinned onto the solid LB agar plates in the absence or presence of the indicated concentration of the drug. The growth phenotypic defect of the strains (i.e., sensitivity or resistance of the mutant strains to the drug) was further examined after two days of incubation at 32 °C.
