# Supplementary material for: Quantitative Genome-Wide Genetic Interaction Screens Reveal Global Epistatic Relationships of Protein Complexes in Escherichia coli
Source: PLoS Genet. 2014 Feb 20;10(2):e1004120. doi: 10.1371/journal.pgen.1004120 (PMC3930520; doi:10.1371/journal.pgen.1004120)
Supplement: Protocol S8 — Effect of Isc pathway overexpression and aminoglycoside treatment on E. coli growth. (PDF) [file pgen.1004120.s013.pdf]

Protocol S8. Effect of Isc pathway overexpression and aminoglycoside treatment on *E. coli* growth

E. coli cells were grown in LB (10 g/L Bacto-tryptone, 5 g/L Yeast extract, and 10 g/L NaCl) at 37 °C with vigorous shaking. For plasmid maintenance, 2.5 μg/mL of tetracycline was added. To generate mild oxidative stress, 3 μg/mL (final concentration) of kanamycin was included in the media essentially as previously described [1]. Growth of the cells was followed by reading OD_{600} at regular time intervals using a SpectraMax M2 Fluorescence Plate Reader. Data represent the average from three independent biological repeats.

References:

1. Kohanski MA, Dwyer DJ, Hayete B, A. LC, Collins JJ (2007) A Common Mechanism of Cellular Death Induced by Bactericidal Antibiotics. Cell 130: 797-810.
