# Supplementary material for: Quantitative Genome-Wide Genetic Interaction Screens Reveal Global Epistatic Relationships of Protein Complexes in Escherichia coli
Source: PLoS Genet. 2014 Feb 20;10(2):e1004120. doi: 10.1371/journal.pgen.1004120 (PMC3930520; doi:10.1371/journal.pgen.1004120)
Supplement: Protocol S9 — Effect of E. coli growth inhibition of RavA overexpression on inorganic and organic sources of sulphur. (PDF) [file pgen.1004120.s014.pdf]

Protocol S9. Effect of *E. coli* growth inhibition of RavA overexpression on inorganic and organic sources of sulphur

E. coli MG1655 wild-type transformed with the plasmid p11 (empty vector control) and pRavA (p11-*ravA*) were grown overnight in LB supplemented with 100 μg/mL ampicillin to prepare the required pre-cultures. On the following day, cells were harvested by centrifugation and were re-suspended in sterilized W-salts media (100 mM potassium phosphate, pH 7.5, 2.1 mM $MgSO_4$, 0.66 μM thiamine, 22 μM glucose, 19 μM NH_4Cl), followed by OD_{600} measurements to assess cell count per unit volume. The re-suspended cells were then added to W-salts media in which sulphur was supplied via 2.1 mM $MgSO_4$, 2.1 mM MgS_2O_3, 0.42 mM taurine, 0.42 mM 2-(4-pyridyl)-ethanesulfonate (PESF) or 0.42 mM cystine (Cys-S-S-Cys). The cell density in all cultures was adjusted to a starting OD_{600} ~0.05. A total of three cultures were prepared for each *E. coli* strain from three independent pre-cultures. Cells were grown at 37 °C, and their growth was monitored by taking OD_{600} measurements at the required time points over 24 hours.
