# Supplementary material for: Quantitative Genome-Wide Genetic Interaction Screens Reveal Global Epistatic Relationships of Protein Complexes in Escherichia coli
Source: PLoS Genet. 2014 Feb 20;10(2):e1004120. doi: 10.1371/journal.pgen.1004120 (PMC3930520; doi:10.1371/journal.pgen.1004120)
Supplement: Protocol S10 — Immunoprecipitation. (PDF) [file pgen.1004120.s015.pdf]

Protocol S10. Immunoprecipitation

The sequential peptide affinity (SPA)-tagged fusion strains with kanamycin selectable marker cassette is endogenously tagged in DY330 by targeted homologous recombination essentially as previously described [1]. Briefly, the sonicated cell lysates from the SPA-tag expressed strains grown at an OD_{600} ~0.6 were incubated for 3 hr at 4°C with anti-Flag M2 agarose beads, which recognizes the FLAG epitope of the SPA-tag. The immunoprecipitated proteins were separated on a 10% SDS polyacrylamide gel and transferred onto nitrocellulose membranes essentially as previously described [1]. The membranes were subsequently probed using the anti-RavA and anti-ViaA polyclonal antibodies generated in rabbits from the University of Toronto, Faculty of Medicine, Division of Comparative Medicine. Antibodies from the sera was purified using CNBr (cyanogen bromide)-activated sepharose beads cross-linked with the respective purified proteins. The membrane probed with the antibodies is visualized by chemiluminescence (Pierce).

References:

1. Babu M, Butland G, Pogoutse O, Li J, Greenblatt JF, et al. (2009) Sequential peptide affinity purification system for the systematic isolation and identification of protein complexes from Escherichia coli. Methods Mol Biol 564: 373-400.
