# Supplementary material for: Quantitative Genome-Wide Genetic Interaction Screens Reveal Global Epistatic Relationships of Protein Complexes in Escherichia coli
Source: PLoS Genet. 2014 Feb 20;10(2):e1004120. doi: 10.1371/journal.pgen.1004120 (PMC3930520; doi:10.1371/journal.pgen.1004120)
Supplement: Protocol S11 — Ribosome profiles, translation fidelity, and cellular RNA analyses. (PDF) [file pgen.1004120.s016.pdf]

Protocol S11. Ribosome profiles, translation fidelity, and cellular RNA analyses

The crude S30 extracts were loaded on linear sucrose density gradients as described [1], and 40% sucrose was used as cushion and ribosomes or subunits were isolated by high-speed ultracentrifugation at 4°C for 16 hrs essentially as previously described [2]. Translational fidelity assay on the deletion mutants were performed with the reported expression plasmids as described earlier [3-5].

The cellular rRNA was analyzed by growing the overnight cultures of the parental WT (BW25113) and the Keio *ΔyaiF* and *ΔrsgA* single gene deletion mutant strains in fresh LB media essentially as previously described [6,7]. The rRNA from the indicated cells was purified using the RNeasy kit (QIAGEN) following manufacturer's protocols. RNA samples were resolved on a 0.7% agarose and 0.9% Synergel for 4h (Diversified Biotech) and processed by Northern hybridization essentially as described [7], except that the primer extension of 16S or 17S rRNAs was performed using a biotnylated oligonucleotide probes. Immunoblot analysis was carried out using HRP-conjugated streptavidin. Single-stranded DNA probe complementary to the internal fragment of 16S rRNA was used as control. The sequences for the probes used are as follows: 16S internal probe (5'-TACTCACCCGTCCGCCACTC-3'); 17S- 5' probe (5'-CGTATCTTCGAGTGCCCACA-3'); 17S- 3' probe (5'- ACGCTTCTTTAAGGTAAGG-3').

References:

1. Jiang M, Sullivan SM, Walker AK, Strahler JR, Andrews PC, et al. (2007) Identification of novel Escherichia coli ribosome-associated proteins using isobaric tags and multidimensional protein identification techniques. J Bacteriol 189: 3434-3444.
2. Campbell TL, Brown ED (2008) Genetic interaction screens with ordered overexpression and deletion clone sets implicate the Escherichia coli GTPase YjeQ in late ribosome biogenesis. J Bacteriol 190: 2537-2545.
3. Babu M, Aoki H, Chowdhury WQ, Gagarinova A, Graham C, et al. (2011) Ribosome-dependent ATPase interacts with conserved membrane protein in Escherichia coli to modulate protein synthesis and oxidative phosphorylation. PLoS One 6: e18510.

4. Hu P, Janga SC, Babu M, Diaz-Mejia JJ, Butland G (2009) Global functional atlas of Escherichia coli encompassing previously uncharacterized proteins. PLoS Biol 7: e1000096.
5. Thompson J, O'Connor M, Mills JA, Dahlberg AE (2002) The protein synthesis inhibitors, oxazolidinones and chloramphenicol, cause extensive translational inaccuracy in vivo. J Mol Biol 322: 273-279.
6. Jomaa A, Stewart G, Martin-Benito J, Zielke R, Campbell TL, et al. (2011) Understanding ribosome assembly: the structure of in vivo assembled immature 30S subunits revealed by cryo-electron microscopy. RNA 17: 697-709.
7. Leong V, Kent M, Jomaa A, Ortega J (2013) Escherichia coli rimM and yjeQ null strains accumulate immature 30S subunits of similar structure and protein complement. RNA 19: 789-802.
