# Supplementary material for: Quantitative Genome-Wide Genetic Interaction Screens Reveal Global Epistatic Relationships of Protein Complexes in Escherichia coli
Source: PLoS Genet. 2014 Feb 20;10(2):e1004120. doi: 10.1371/journal.pgen.1004120 (PMC3930520; doi:10.1371/journal.pgen.1004120)
Supplement: Protocol S12 — Computing correlation and determining optimal cut-off score for deriving GI profiles. (PDF) [file pgen.1004120.s017.pdf]

Protocol S12. Computing correlation and determining optimal cut-off score for deriving GI profiles

To identify GI profiles for each gene pair [1], we computed the Pearson correlation coefficient (PCC) for each donor and recipient gene with the rest of the genes. For assessing an optimal correlation cut-off value, we applied a precision- recall curve to determine how often the correlated GI pair belonging to the same pathway is recapitulated compared to a random dataset. We addressed this by defining a true positive (TP) pair for gene pairs sharing a common Ecocyc complex or pathway [2], and a false positive (FP) for all other gene pairs. Recall is defined as fraction of positives recovered at a certain cut-off (TP/FP), while precision is calculated as TP / (TP+FP). The precision curves were plotted using the ROCR package [3], and an optimal cut-off score of PCC ≥ 0.3 was chosen based on the precision-recall curve (i.e., precision of ~0.07 and a recall of ~0.2 among 2,385 EcoCyc complex or pathway gene pairs tested), which tend to be higher than random (i.e., pathway annotations randomized 1,000 times by shuffling the gene labels, while retaining the frequency for each gene annotation).

References:

1. Costanzo M, Baryshnikova A, Bellay J, Kim Y, Spear ED, et al. (2010) The genetic landscape of a cell. Science 327: 425-431.
2. Keseler IM, Collado-Vides J, Santos-Zavaleta A, Peralta-Gil M, Gama-Castro S, et al. (2011) EcoCyc: a comprehensive database of Escherichia coli biology. Nucleic Acids Res 39: D583-590.
3. Sing T, Sander O, Beerenwinkel N, Lengauer T (2005) ROCR: visualizing classifier performance in R. Bioinformatics 21: 3940-3941.
