# Supplementary material for: Quantitative Genome-Wide Genetic Interaction Screens Reveal Global Epistatic Relationships of Protein Complexes in Escherichia coli
Source: PLoS Genet. 2014 Feb 20;10(2):e1004120. doi: 10.1371/journal.pgen.1004120 (PMC3930520; doi:10.1371/journal.pgen.1004120)
Supplement: Protocol S13 — Analysis on chaperone complexes and their association with epistatic interactions. (PDF) [file pgen.1004120.s018.pdf]

Protocol S13. Analysis on chaperone complexes and their association with epistatic interactions

To examine how many chaperone-containing protein complexes are enriched genetically with maximum number of other chaperones, we computed the probability mass function of the hypergeometric distribution for 18 protein complexes, encompassing 16 chaperone proteins, compiled from EcoCyc [1] and Hu et al [2] using the following equation:

$$P\ (X = x) = h\ (x; n, M, N) = \frac{\binom{M}{x}\binom{N-M}{n-x}}{\binom{N}{n}}$$

Where: x = number of chaperone proteins interacting with a complex; n = number of chaperone proteins present in all protein complexes; M = total verified chaperone proteins in *E.* coli, and N = total number proteins in *E. coli*. Based on this analysis, 18 chaperone protein complexes tend to be enriched significantly (*p*-value ≤ 0.05) for GIs with chaperone proteins, which was subsequently used to generate the distribution plot.

To assess the overlap of non-chaperone interactors among the 21 chaperone containing protein complexes, the following Jaccard Index equation was employed:

$$\text{Jaccard Index} = \frac{(\text{interactors of complex A} \cap \text{interactors of complex B})}{(\text{interactors of complex A} \cup \text{interactors of complex B})}$$

References:

1. Keseler IM, Collado-Vides J, Santos-Zavaleta A, Peralta-Gil M, Gama-Castro S, et al. (2011) EcoCyc: a comprehensive database of Escherichia coli biology. Nucleic Acids Res 39: D583-590.
2. Hu P, Janga SC, Babu M, Diaz-Mejia JJ, Butland G (2009) Global functional atlas of Escherichia coli encompassing previously uncharacterized proteins. PLoS Biol 7: e1000096.
