# Supplementary material for: Quantitative Genome-Wide Genetic Interaction Screens Reveal Global Epistatic Relationships of Protein Complexes in Escherichia coli
Source: PLoS Genet. 2014 Feb 20;10(2):e1004120. doi: 10.1371/journal.pgen.1004120 (PMC3930520; doi:10.1371/journal.pgen.1004120)
Supplement: Protocol S14 — eSGA inter-module permutation test. (PDF) [file pgen.1004120.s019.pdf]

Protocol S14. eSGA inter-module permutation test

We mapped 42,705 putative digenic interactions from our eSGA screen with *S*-scores $|\geq 3|$ at a threshold of 2 standard deviations of significance onto a previously published set of putative *E. coli* functional module predictions [1,2], resulting in a combined functional module-GI network. Two classes of GI were investigated, those occurring within the same functional module (i.e., intra-module GIs), and those between distinct functional module pairs (i.e., inter-module GIs). To assess the significance of these interacting module pairs, we implemented a permutation test to determine those which appeared to be enriched in GIs compared to a random background distribution, which would suggest possible connections of biological relevance for further exploration.

The permutation test was performed as follows. Briefly, we randomly exchanged interaction partners for each gene participating in an intra- or inter-module GI. After randomizing every interacting gene pair, the total number of intra- and inter-module GIs found between functional module pairs was recalculated. This procedure was repeated for 1,000 iterations, after which we calculated the average and standard deviation of intra- and inter-module GIs for each functional module pair of the randomized network. We then compared the number of intra- and inter-module interactions between functional modules of the original dataset with the randomized network using a Z-Score. Intra- and inter-module pairs with Z-scores ≥ 2.5 (p-value ≤ 0.05) were chosen for further analysis.

References:

1. Hu P, Janga SC, Babu M, Diaz-Mejia JJ, Butland G (2009) Global functional atlas of Escherichia coli encompassing previously uncharacterized proteins. PLoS Biol 7: e1000096.
2. Peregrin-Alvarez JM, Xiong X, Su C, Parkinson J (2009) The Modular Organization of Protein Interactions in Escherichia coli. PLoS Comput Biol 5: e1000523.
