# Supplementary material for: Quantitative Genome-Wide Genetic Interaction Screens Reveal Global Epistatic Relationships of Protein Complexes in Escherichia coli
Source: PLoS Genet. 2014 Feb 20;10(2):e1004120. doi: 10.1371/journal.pgen.1004120 (PMC3930520; doi:10.1371/journal.pgen.1004120)
Supplement: Protocol S15 — Generation of phylogenetic co-occurrence using correlated GI profiles and MI scores. (PDF) [file pgen.1004120.s020.pdf]

Protocol S15. Generation of phylogenetic co-occurrence using correlated GI profiles and MI scores

Orthology or paralogy predictions of the protein-coding sequences of recipient *E. coli* genes with the corresponding PCC correlation of GI profiles (i.e., between all possible donor and recipient gene pairs) were downloaded and extracted from the eggNOG online database ver. 3.0 [1] for 233 fully sequenced γ-proteobacterial species (γ-proteobacterial NOGs). The 16S rRNA nucleotide sequences from these 233 γ-proteobacterial species were extracted from the NCBI genome database (downloaded as of September, 2012) and a general phylogenetic ordering relative to the *E. coli* W3110 laboratory strain was determined using a BlastN search from the Blast+ suite [2]. The list of 233 γ-proteobacterial species and their 16S rRNA percentage identity values relative to *E. coli* W3110 are shown in Table S14.

Phylogenetic profiles were generated for the presence of single or multiple bacterial protein sequences as possible hits for each recipient gene of the eSGA screen across 233 γ-proteobacterial species. For all recipient gene pairs having correlated GI profiles with PCC ≥ 0.5, the similarity between their patterns of co-conservation was calculated using mutual information (MI) score [3], according to the following equation:

$$MI = \sum_{i,j}^{N} Pij(A,B)\log \frac{Pij\ (A,B)}{Pi(A)Pj(B)}$$

Where, A and B represent the phylogenetic profiles of a donor and recipient gene, respectively; N indicates the “state” of a gene in a genome [i.e., presence (i, j = 1), or absence (i, j = 0)]. Therefore for a pair of genes, four possible states (N=4), or co-conservation patterns emerge: (i) when genes A and B are mutually present in a given genome; (ii) when genes A and B are mutually absent; (iii) when gene A is present but B is absent; and (iv) vice-versa. Therefore, p_i

(A) and p_j (B) represents the frequency of gene presence or absence of gene A and B, across all genomes of their respective phylogenetic profiles, while $p_{i,j}$ (A, B) represents the frequency of each of the four co-conservation patterns as described above. It is important to note that in the calculation of gene co-conservation using MI, we only considered the binary state of either the presence or absence of a given *E. coli* recipient gene in a γ-proteobacterial genome, and do not take into account whether a gene may be present in multiple copies.

Phylogenetic co-occurrence of all possible gene pairs was computed, resulting in a set of 1,732,591 non-redundant non-zero MI scores (mean = 0.0384, 1-sided 5% cut-off = 0.1033), which we used for further analysis. Many PCC interactions were excluded from co-conservation analysis due to uninformative phylogenetic profiles (i.e., profiles where one or both gene pairs are present), resulting in MI score of 0.

References:

1. Powell S, Szklarczyk D, Trachana K, Roth A, Kuhn M, et al. (2012) eggNOG v3.0: orthologous groups covering 1133 organisms at 41 different taxonomic ranges. Nucleic Acids Res 40: D284-289.
2. Camacho C, Coulouris G, Avagyan V, Ma N, Papadopoulos J, et al. (2009) BLAST+: architecture and applications. BMC Bioinformatics 10: 421.
3. Wu J, Kasif S, DeLisi C (2003) Identification of functional links between genes using phylogenetic profiles. Bioinformatics 19: 1524-1530.
