# Supplementary material for: Quantitative Genome-Wide Genetic Interaction Screens Reveal Global Epistatic Relationships of Protein Complexes in Escherichia coli
Source: PLoS Genet. 2014 Feb 20;10(2):e1004120. doi: 10.1371/journal.pgen.1004120 (PMC3930520; doi:10.1371/journal.pgen.1004120)
Supplement: Protocol S16 — Bacterial strains, plasmids, and genetic screens. (PDF) [file pgen.1004120.s021.pdf]

Protocol S16. Bacterial strains, plasmids, and genetic screens

The F- 'recipient' single gene deletion knock-out strain marked with Kan^R were from the Keio mutant library [1]. The Hfr C non-essential donor gene deletion mutant strains or essential gene hypomorphic mutations were constructed using the λ-Red recombination [2-4] or P1 phage transduction [5] system. For assessing the effect of *ravA* and *viaA* on oxidative stress, we used the plasmids pRKISC and pRKNMC donated by Prof. Yasuhiro Takahashi (Saitama University, Japan). pRKISC carries the entire *isc* gene cluster (*iscRSUA-hscBA-fdx-iscX*) and has been shown to enhance the biogenesis of Fe-S clusters [6], whereas pRKNMC served as the empty vector control for pRKISC. Both the wild type (WT) and *ravAviaA* mutants were transformed with the aforementioned two plasmids to obtain WT-pRKISC, WT-pRKNMC, Δ*ravAviaA*-pRKISC, and Δ*ravAviaA*-pRKNMC strains.

References:

1. Baba T, Ara T, Hasegawa M, Takai Y, Okumura Y, et al. (2006) Construction of Escherichia coli K-12 in-frame, single-gene knockout mutants: the Keio collection. Mol Syst Biol 2: 2006.0008.
2. Babu M, Díaz-Mejía JJ, Vlasblom J, Gagarinova A, Phanse S, et al. (2011) Genetic interaction maps in Escherichia coli reveal functional crosstalk among cell envelope biogenesis pathways. PLoS Genet 7: e1002377.
3. Babu M, Gagarinova A, Greenblatt J, Emili A (2011) Array-based synthetic genetic screens to map bacterial pathways and functional networks in Escherichia coli. Methods Mol Biol 765: 125-153.
4. Butland G, Babu M, Díaz-Mejía JJ, Bohdana F, Phanse S, et al. (2008) eSGA: E. coli synthetic genetic array analysis. Nat Methods 5: 789-795.
5. Miller JH (1992) A short course in bacterial genetics Miller JH, editor. Plainview, NY: Cold Spring Harbor Laboratory Press
6.Takahashi Y, Nakamura M (1999) Functional Assignment of the ORF2-*iscS*-*iscU*-*iscA*-*hscB*-*hscA*-*fdx*-ORF3 Gene Cluster Involved in the Assembly of the Fe-S Clusters in *Escherichia coli*. J Biochem 126: 917-926.
